# Monitoring nucleolar-nucleoplasmic protein shuttling in living cells by high-content microscopy and automated image analysis

**DOI:** 10.1093/nar/gkae598

**Published:** 2024-07-22

**Authors:** Marina Engbrecht, David Grundei, Asisa M Dilger, Hannah Wiedemann, Ann-Kristin Aust, Sarah Baumgärtner, Stefan Helfrich, Felix Kergl-Räpple, Alexander Bürkle, Aswin Mangerich

**Affiliations:** Molecular Toxicology, Department of Biology, University of Konstanz, 78457 Konstanz, Germany; Molecular Toxicology, Department of Biology, University of Konstanz, 78457 Konstanz, Germany; Nutritional Toxicology, Institute of Nutritional Science, University of Potsdam, 14469 Potsdam, Germany; Molecular Toxicology, Department of Biology, University of Konstanz, 78457 Konstanz, Germany; Molecular Toxicology, Department of Biology, University of Konstanz, 78457 Konstanz, Germany; Molecular Toxicology, Department of Biology, University of Konstanz, 78457 Konstanz, Germany; KNIME GmbH, Reichenaustr. 11, 78467 Konstanz, Germany; KNIME GmbH, Reichenaustr. 11, 78467 Konstanz, Germany; Molecular Toxicology, Department of Biology, University of Konstanz, 78457 Konstanz, Germany; Molecular Toxicology, Department of Biology, University of Konstanz, 78457 Konstanz, Germany; Nutritional Toxicology, Institute of Nutritional Science, University of Potsdam, 14469 Potsdam, Germany

## Abstract

The nucleolus has core functions in ribosome biosynthesis, but also acts as a regulatory hub in a plethora of non-canonical processes, including cellular stress. Upon DNA damage, several DNA repair factors shuttle between the nucleolus and the nucleoplasm. Yet, the molecular mechanisms underlying such spatio-temporal protein dynamics remain to be deciphered. Here, we present a novel imaging platform to investigate nucleolar-nucleoplasmic protein shuttling in living cells. For image acquisition, we used a commercially available automated fluorescence microscope and for image analysis, we developed a KNIME workflow with implementation of machine learning-based tools. We validated the method with different nucleolar proteins, i.e., PARP1, TARG1 and APE1, by monitoring their shuttling dynamics upon oxidative stress. As a paradigm, we analyzed PARP1 shuttling upon H_2_O_2_ treatment in combination with a range of pharmacological inhibitors in a novel reporter cell line. These experiments revealed that inhibition of SIRT7 results in a loss of nucleolar PARP1 localization. Finally, we unraveled specific differences in PARP1 shuttling dynamics after co-treatment with H_2_O_2_ and different clinical PARP inhibitors. Collectively, this work delineates a highly sensitive and versatile bioimaging platform to investigate swift nucleolar-nucleoplasmic protein shuttling in living cells, which can be employed for pharmacological screening and in-depth mechanistic analyses.

## Graphical Abstract

**Graphical Abstract:**
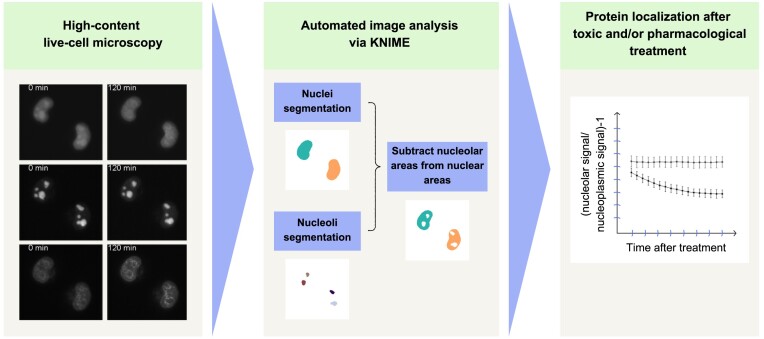

## Introduction

Nucleoli are membrane-less, subnuclear compartments, best known for their function in ribosome biogenesis [reviewed in (1)]. In addition, the nucleolus was identified as a central hub in regulating a plethora of other cellular processes, such as cell cycle control, cell proliferation, stress response and DNA damage signaling [reviewed in (2,3)]. Consistently, nucleolar dysfunctions were shown to be implicated in the development of severe human diseases, most importantly in the formation of various types of cancer [reviewed in (4)]. Recently the nucleolus has emerged as a promising novel target in cancer therapy [reviewed in (5)]. Nucleoli assemble around tandemly repeated clusters of rDNA genes, called nucleolar organizing regions (NORs), which are located on the short arms of the five human acrocentric chromosomes (6,7). The transcriptionally active genes are surrounded by a shell of densely packed DNA, termed peri-nucleolar heterochromatin (PNH). The PNH constitutes a major part of so-called nucleolus-associated domains (NADs), which dynamically interact with nucleoli [reviewed in (8)]. Mammalian nucleoli typically display a tri-partite organization, which is associated with ongoing rDNA transcriptional processes, consisting of fibrillar centers (FCs), dense fibrillar components (DFCs) and the granular component (GC) [reviewed in (9)]. Throughout the cell cycle or in response to cellular stress, nucleoli undergo dynamic reorganization, with many proteins entering or exiting nucleoli. Of note, under non-stress conditions more than 150 DNA repair proteins are sequestered in nucleoli [reviewed in (10)]. At least some of these DNA repair proteins undergo nucleolar-nucleoplasmic shuttling upon certain stimuli of genotoxic stress [(11) and reviewed in (12)].

One of these proteins is poly(ADP-ribose) polymerase 1 (PARP1), also known as ADP-ribosyltransferase diphtheria toxin-like 1 (ARTD1), which belongs to the PARP family comprising 17 members in humans [reviewed in (13,14)]. By using nicotinamide adenine dinucleotide (NAD^+^) as a substrate several PARPs catalyze ADP-ribosylation, a highly dynamic and fully reversible post-translational modification (PTM), which can be found in most eukaryotes (15). While some PARP family members are catalytically inactive or transfer only a single ADP-ribose unit to acceptor proteins (monoADP-ribosylation, MARylation), PARP1, PARP2 and tankyrases (i.e. TNKS1 and TNKS2) are known to synthesize polymers of ADP-ribose (PAR) in a process called poly(ADP-ribosyl)ation (PARylation) [reviewed in (13,14)]. The negatively charged PAR chains can be either linear or branched with varying branching frequencies and reach a length of up to 200 ADP-ribose units [reviewed in (16)].

PARP1 becomes mainly activated by DNA single or double strand breaks and may account for up to 90% of DNA damage-induced PARylation (17,18). In addition to its critical role in DNA damage response and genome maintenance, it exhibits crucial functions in other cellular processes, including chromatin remodeling, transcription, cell cycle control and cell death [reviewed in (19)]. Moreover, PARP1 has been implicated in a range of pathophysiological conditions, such as carcinogenesis, inflammation, diabetes, cardiovascular and neurodegenerative diseases [reviewed in (20)]. For this reason, the therapeutic potential of PARP inhibitors (PARPi) was extensively studied, resulting in the clinical approval of several PARPi for the treatment of different types of cancers [reviewed in (20,21)].

Under non-stress conditions, around 40% of cellular PARP1 resides in nucleoli, where it was reported to play important roles in nucleolar biology [(22) and reviewed in (23)]. Upon DNA damage induction or inhibition of RNA polymerase I (Pol I), PARP1 can translocate into the nucleoplasm (22,24,25). In a previous study, we demonstrated that H_2_O_2_ treatment induces PARP1 shuttling from nucleoli into the nucleoplasm (26). Despite substantial research, the exact molecular mechanisms underlying the DNA damage-induced nucleolar-nucleoplasmic shuttling of proteins remain elusive [reviewed in (23)]. In general, such protein dynamics are assumed to be regulated by a network of PTMs, such as acetylation, phosphorylation, and ubiquitination, as well as by protein-protein interactions [(27–30) and reviewed in (10)]. Our previous study revealed a prominent role of PARP1 and PARylation activity in regulating the DNA damage-induced nucleolar-nucleoplasmic shuttling of two important genome maintenance factors, i.e. the RecQ helicase WRN and the base excision repair protein XRCC1 (26). Importantly, the mechanisms underlying nucleolar-nucleoplasmic shuttling of proteins appear to be highly protein- and toxicant-specific, which adds another layer of complexity to the topic.

Several studies investigating stress-induced nucleolar-nucleoplasmic protein shuttling were conducted by analyzing fixed and stained cells (31–36), including our own previous research (26). While these studies provided important insights, using fixed cells comes along with the disadvantage that only static snapshots of the cell state are revealed.

In the present study, we used high-content microscopy in combination with a newly developed automated, machine learning-based KNIME (37) workflow to monitor and analyze stress-induced nucleolar-nucleoplasmic protein shuttling in living cells. We validated the analysis tool by using a transient transfection approach of various expression constructs coding for eGFP-tagged nucleolar proteins. Furthermore, a HeLa reporter cell line stably expressing PARP1-eGFP and the nucleolar marker NPM1-mCherry was generated, which was used to perform detailed analyses of PARP1 shuttling dynamics upon treatment with H_2_O_2_ alone or in combination with different pharmacological inhibitors, including clinically relevant PARPi.

## Materials and methods

### Cell culture, transient transfection and genotoxic treatment

HeLa WT and stably transfected HeLa ‘PARP1-eGFP + NPM1-mCherry’ cells were cultured at 37°C, 5% CO_2_ and 95% humidity in DMEM (Gibco) supplemented with 10% fetal bovine serum (FBS, Biochrom), 1% penicillin/streptomycin (Gibco) and 2 mM glutamine (Gibco). HeLa ‘PARP1-eGFP + NPM1-mCherry’ cells were cultured in the presence of 400 μg/ml G418 (Sigma) to maintain antibiotic selection pressure. For transient transfections with PARP1-eGFP, eGFP-TARG1 (kind gift of B. Lüscher, RWTH Aachen University) (48), APE1-eGFP (kind gift of G. Tell, University of Udine) and NPM1-mCherry (kind gift of E. Ferrando-May, Bioimaging Center, University of Konstanz), cells were seeded at a density of 3 × 10^5^ per well in 6-well plates. Transient transfection of HeLa WT cells was performed 24 h after the seeding using 3.75 μl Lipofectamine 3000 transfection reagent (Thermo Fisher Scientific) according to the manufacturer's instructions. The medium was exchanged 6 h after transfection and live-cell experiments were performed one or two days later.

### Generation of HeLa cells stably expressing PARP1-eGFP and NPM1-mCherry

For stable transfection, 3 × 10^4^*PARP1* KO cells (18) per well were seeded in 6-well plates and transfected 24 h later with linearized PARP1-eGFP and NPM1-mCherry constructs as described above. The next day, cells were passaged at a ratio of 1:10 and then incubated for 24 h to allow them to attach on plates. Selection medium was added to the cells 48 h post-transfection, using the concentration of 800 μg/ml G418 as determined beforehand in a kill curve. The selective medium was renewed every 2–3 days until a resistant pool of cells was obtained. To isolate individual clones single cell sorting was performed at the flow cytometry facility FlowKon at the University of Konstanz. These clones were expanded and evaluated using FACS analysis and immunofluorescence microscopy.

### Western blot analysis and protein expression

Cells were seeded in 6-well plates at a density of 3 × 10^5^ cells per well. After 24 h cells were treated with 0, 50 or 500 μM H_2_O_2_ [30% (w/w), Sigma] for 120 min. Subsequently, cells were harvested by scraping in lysis buffer containing 20 mM Tris–HCl, 150 mM NaCl, 1 mM EDTA, 1 mM EGTA, 1% (v/v) Triton X-100, 2.5 M sodium pyrophosphate, ß-glycerolphosphate and 50 mM sodium fluoride, followed by centrifugation at 400 × *g* for 15 min at 4°C. Supernatants were collected and either frozen at −20°C or directly subjected to a BCA assay to determine total protein content. Samples were adjusted to 1 μg/μl in 5× SDS sample buffer and ddH_2_O, heated at 95°C for 5 min, and stored at −20°C until further use. For SDS-PAGE, 30 μg of protein was loaded onto a 10% polyacrylamid gel and electrophoresis was performed at 100 V for 1 h. Western blotting was performed on either a 0.45-μm nitrocellulose or PVDF membrane (both from Carl Roth) using 5% milk in TBST [50 mM Tris base pH 7.5, 150 mM NaCl, 0.1% (v/v) Tween20] as a blocking solution. Primary antibodies, i.e. mouse-anti-PARP1 (CII10, purified from culture supernatant of hybridoma cells, 1:300), mouse-anti-NPM1 (1:1000, Proteintech), and rabbit-anti-COX-IV (1:1000, Cell Signaling), were incubated overnight at 4°C. After washing in TBST, membranes were incubated for 1 h with the respective secondary antibodies, i.e. goat-anti-mouse HRP-coupled or goat-anti-rabbit HRP-coupled (both 1:2500, Proteintech). Membranes were imaged using SuperSignal West Pico PLUS chemiluminescent substrate (ThermoScientific) according to the manufacturer's instructions on a ChemiDoc XRS+ imaging station (BioRad). Densitometric analysis was performed using Fiji software.

### Immunofluorescence staining for poly(ADP-ribose) and confocal microscopy

For immunofluorescence staining, HeLa WT and ‘PARP1-eGFP + NPM1-mCherry’ cells were seeded on 15-mm coverslips at a density of 1 × 10^5^ cells per well in 12-well plates. After 24 h, cells were treated as indicated and subsequently washed once in PBS. Fixation was performed in a fresh 12-well plate by incubating cells for 20 min in 4% PFA in PBS, followed by 1 min incubation with 100 mM glycine in PBS to stop the reaction. After washing once with PBS, cells were permeabilized for 3 min in 0.4% Triton X-100 in PBS, followed by another washing step for 5 min. To prevent unspecific antibody binding, samples were incubated for 1 h at RT in PBSM-T [5% (w/v) non-fat dry milk, 0.5% Tween20 in PBS]. Then, cells were incubated with the primary mouse-anti-PAR antibody (10H, 1:300 in PBSM-T) for 1 h at RT or at 4°C overnight. Afterwards, cells were washed 3 × 10 min in PBS and then incubated with the secondary antibody goat-anti-mouse IgG coupled to Alexa Fluor 647 (Invitrogen, 1:400 in PBSM-T) for 1 h at RT in the dark. Then cells were washed 3 × 10 min in PBS and samples were incubated with 0.2 μg/ml Hoechst33342 in PBS for 5 min to counterstain nuclei. Cells were again washed in 3 × 5 min in PBS and coverslips were mounted on microscopy slides using Aqua Polymount (Polysciences). Thereafter, slides were stored for at least 24 h to allow solidification of Aqua Polymount and analyzed using confocal laser scanning microscopy.

Confocal images were acquired using a Zeiss LSM700 confocal microscope at the Bioimaging Center of the University of Konstanz. Samples were analyzed with a Plan-Apochromat 63×/1.4 Oil Ph3 M27 objective. Hoechst33342 was excited with a 405-nm diode, eGFP with a 488-nm diode, mCherry with a 555-nm diode and Alexa Fluor 647 with a 637-nm diode. The pinhole of the channel with the highest wavelength was adjusted to 1 airy unit (AU) and the resulting pinhole diameter was adjusted to the same value in the remaining channels. For each condition at least five images were acquired with a resolution of 1440 × 1440 pixels. For better visibility linear adjustments of brightness and contrast were performed using Fiji software.

### Live-cell imaging

For live-cell imaging, 3 × 10^4^ cells were seeded in black ibiTreat 96-well plates (ibidi) in 300 μl DMEM. At least 3 h before image acquisition, the medium was aspirated, cells were washed once with pre-warmed DMEM and, for nuclear staining, phenol red-free DMEM (Gibco) containing 50 nM SiR-Hoechst and 10 μM of the efflux inhibitor verapamil were added to the cells. Live-cell imaging was performed in a controlled environment (37°C, 5% CO_2_) using the automated imaging system Celldiscoverer 7 (Zeiss). A Plan-Apochromat 20x/0.95 objective with a 2x tube lens and the integrated Axiocam 506 mono-camera were used for image acquisition. SiR-Hoechst was excited using a 650-nm LED, mCherry a 567-nm LED and eGFP a 509-nm LED. Live-cell imaging in 2D was performed for 120–180 min in 5 min intervals using the ‘*Definite Focus*’ function for focus stabilization. For better visibility of presented imaging data, linear adjustments of brightness and contrast were performed using Fiji. Usually, per well five images were acquired containing typically between 20 and 50 cells (Supplementary Figure S1).

### Manual image analysis using Fiji

Intensity profiles shown in Figure 3 were obtained using Fiji and GraphPad Prism. For each condition, 10 cells were analyzed. First, a line was drawn across the nucleus and the center of one nucleolus within a cell by using the line tool in Fiji. By running the < Analyze > and < Plot Profile > tools, intensity line plots were generated for each single channel. To ensure that the position of the line was equal in all single channels, Fiji's ROI Manager was used. Applying the ‘List’ function produced tables containing the plot values, which were then exported to GraphPad Prism. To compensate for different expression levels of the transfected plasmids the values were normalized based on the maximal gray values at 0 and 120 min.

To analyze the SiR-Hoechst intensity the maximal values of the SiR-Hoechst channel at 0 min were set to 100%. For the quantification of the change in SiR-Hoechst intensity over time the areas under the curve (AUC) of the normalized intensity profiles at 0 and 120 min were calculated, then the AUC at 120 min were divided by the AUC at 0 min.

### Automated image analysis using KNIME

For the automated analysis of live-cell imaging data acquired at the Celldiscoverer 7 system, a workflow was developed as described in detail in the Results section using KNIME. The Konstanz Information Miner (KNIME) Analytics Platform is an open-source software enabling users to create tailored data analysis workflows without requiring programming skills (37), which is accessible through the KNIME homepage (https://www.knime.com/). Usually, five microscopic images containing typically between 20–50 cells were evaluated per well (Supplementary Figure S1). The workflow started with loading and reading of files, which was followed by data pre-processing, segmentation and finally feature extraction. The pipeline started with the ‘*List Files’* node, which was used to choose a folder containing the images to be analyzed. After several path manipulation steps, which were encapsulated in the ‘*File Path Manipulation’* meta node, the multichannel images were read in using the ‘*Image Reader’* node. During data pre-processing, the ‘*Splitter’* node was used to split the multi-channel images into single channels. Subsequently, the SiR-Hoechst channel was used for segmentation of nuclei and the NPM1-mCherry channel for the segmentation of nucleoli. For the segmentation of nuclei, StarDist (38), which is a deep learning tool that uses star-convex polygons for detection of nuclei, was implemented into the workflow. For the segmentation of nucleoli, a model was pre-trained in Ilastik (39), which was included into the workflow via the ‘*Ilastik Headless (Pixel Classification)’* node. To define nucleoplasmic regions, nucleolar regions were subtracted from nuclear regions by using the ‘*Labeling Arithmetic’* node. In the ‘*Feature Extraction’* meta node nucleolar and nucleoplasmic fluorescence intensities of the protein of interest were measured using the ‘*Image Segment Features’* node, then ratios of the mean nucleolar and nucleoplasmic fluorescence intensities were calculated for each time point using the ‘*Math Formula’* node. For better illustration a value of ‘1’ was subtracted from the ratios. Finally, the obtained results were saved in an Excel spreadsheet using the ‘*Excel Writer’* node.

For the automated analysis of the SiR-Hoechst intensity the results of the nuclei segmentation were used to define nuclear regions. Using the ‘*Image Segment Features’* node, the mean SiR-Hoechst intensities in the nuclear regions were measured. Afterwards, the mean SiR-Hoechst intensities were calculated for each condition and each time point using the ‘*Math Formula’* node. The complete KNIME workflow is available on KNIME Community Hub (https://hub.knime.com/-/spaces/-/latest/∼6kcnnzQRlDlyc1GO/).

## Results

### Development of an imaging platform to monitor nucleolar-nucleoplasmic protein shuttling in living cells

High-content live-cell fluorescence microscopy allows to automatically monitor dynamic cellular processes in real-time at high-throughput, thereby producing significant amounts of imaging data. In this study, we used an automated live-cell fluorescence imaging system, i.e. the Celldiscoverer 7 (Zeiss). We performed triple-color fluorescence microscopy at multiple pre-selected positions at 5 min intervals, enabling the monitoring of stress-induced nucleolar-nucleoplasmic protein shuttling in living cells. To accurately and efficiently analyze the acquired images, we used the KNIME Analytics Platform, which allows users to build customized data analysis workflows without the need of programming expertise [see Materials and methods section] (37). This is achieved through the combination and configuration of different nodes, which represent the ‘atomic processing units’ in KNIME (40). The ‘*KNIME Image Processing (KNIP)’* extension available within the KNIME Analytics Platform enables the generation of reproducible image analysis workflows. Such workflows can be combined with ImageJ functionalities and other available extensions, such as machine learning, statistical analysis, and data visualization (41).

**Figure 1.**
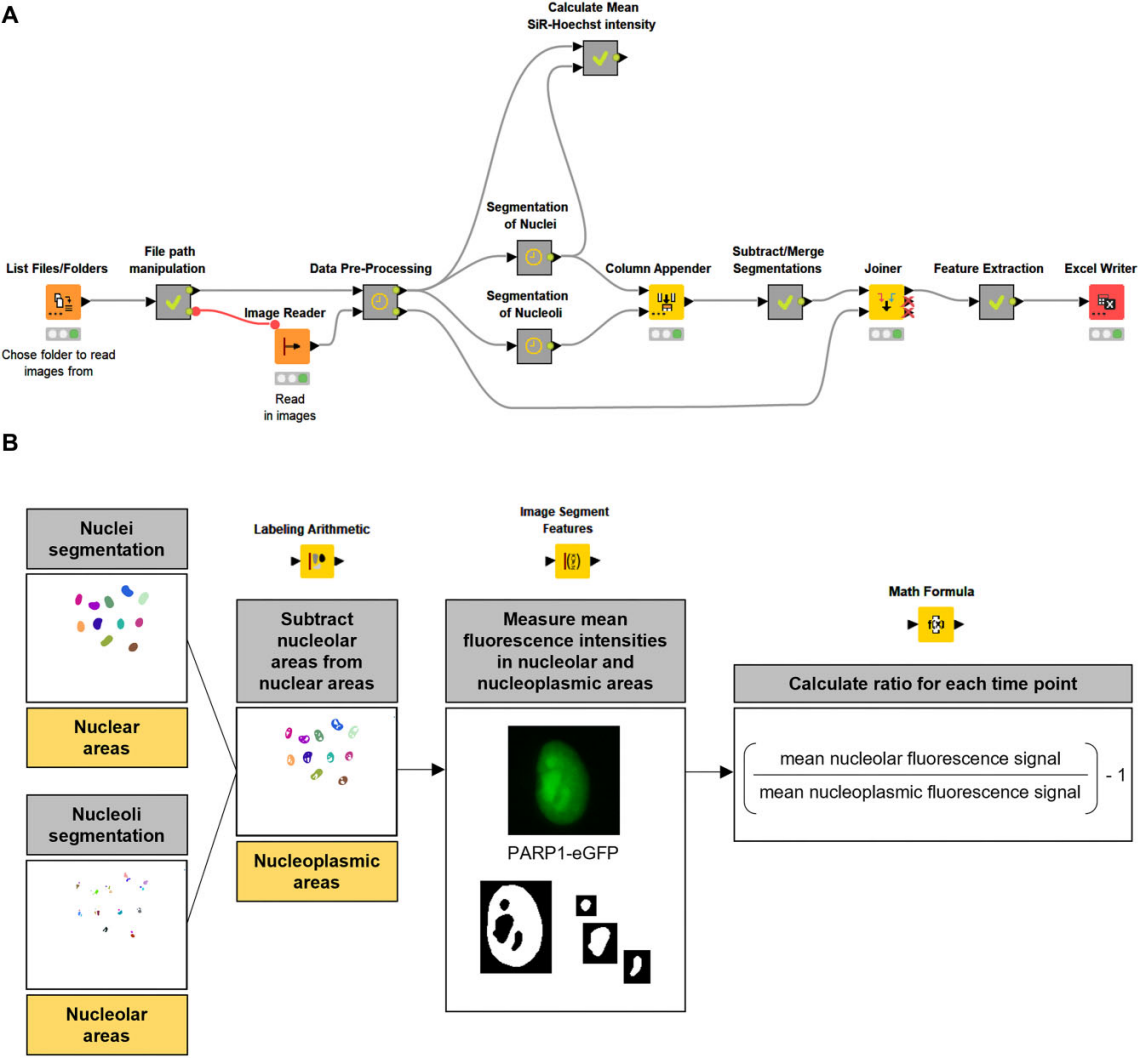
Development of a KNIME workflow for automated analysis of high-content live-cell imaging data of nucleolar-nucleoplasmic protein shuttling dynamics. (**A**) Overview of the workflow. Grey nodes represent meta nodes, which encapsulate sub-workflows. (**B**) Illustration of individual steps. For details see text. The complete KNIME workflow is available on KNIME Community Hub (https://hub.knime.com/-/spaces/-/latest/∼6kcnnzQRlDlyc1GO/).

To set up the new image analysis workflow, as a starting point, we used a workflow that was formerly developed in our group for the analysis of subnuclear protein localizations in fixed cells as a basic framework (26). Figure 1A shows an overview of the newly developed workflow. The grey nodes shown in Figure 1A are meta nodes that encapsulate subparts of the workflow, which were omitted for reasons of space and clarity. The complete KNIME workflow is available on KNIME Community Hub (https://hub.knime.com/-/spaces/-/latest/∼6kcnnzQRlDlyc1GO/). Before using this workflow, we applied the ‘*Split Scenes (Write Files)*’ function in the Zeiss ZEN blue user interface on our acquired data to obtain separate files, of which each included the image sequences of one imaging position. Subsequently, the files were re-organized in separate folders, each containing images of one experimental condition. The first node ‘*List Files’* was used to choose a folder containing the images to be analyzed. By default, multi-channel live-cell images with various file formats supported by the ‘Bio-Formats’ library can be loaded into the workflow. The imaging data were saved as ‘.czi’ files and contained information on meta data, such as the corresponding scene number, position number and well designation in their file names. These file naming conventions were also used in the ‘File Path Manipulation’ meta node and should therefore be taken into account when naming files. The ‘*Image Reader’* node, which supports images from various file formats, was used to read in and convert images into a format compatible with KNIME. Subsequently, the multi-channel images were split into single channels by applying the ‘*Splitter’* node. In our experiments we used the (NPM1-)mCherry channel for nucleoli segmentation to define nucleolar regions, while the (SiR-)Hoechst channel was used for nuclei segmentation. NPM1 is a well-established marker protein for nucleoli, which localizes to the granular component (42) and has been used for nucleolar region detection throughout this study. SiR-Hoechst is a cell-permeable, far-red fluorescence dye widely utilized in live-cell microscopy. In this study, it was used at concentrations demonstrated to be non-cytotoxic and non-genotoxic (43,44). The meta nodes for image segmentation represent the core of this workflow and are therefore described in more detail: To determine nucleoplasmic regions, nucleolar regions were subtracted from nuclear regions by using the ‘*Arithmetic Labeling’* node as illustrated in Figure 1B. In addition, a second ‘*Arithmetic Labeling’* node was included, in which nuclear and nucleolar regions were merged, to assign each nucleolus to the corresponding nucleus. In the ‘*Feature Extraction’* meta node, the mean fluorescence intensities of the protein of interest in nucleolar as well as nucleoplasmic regions were measured using the ‘*Image Segment Features’* node, then the ratios of nucleolar and nucleoplasmic fluorescence intensities were calculated for each time point. For better illustration, the value of ‘1’ was subtracted from the ratios. Finally, in the ‘*Excel Writer’* node the calculated ratios and the corresponding time points (‘iteration’) were exported into a spreadsheet of an Excel file, which was subsequently used for downstream data processing. Examples of images from two microscopic positions of a novel HeLa ‘PARP1-eGFP + NPM1-mCherry’ reporter cell line (for more information on the cell line see below) are shown in Supplementary Figure S1. Usually, for each experimental condition, five microscopic images were acquired per well, each containing typically 20–50 cells. In total, normally, this led to the analysis of at least 100 cells per condition. In summary, we developed a KNIME workflow, which computes ratios of nucleolar and nucleoplasmic fluorescence signals for each time point, thereby allowing to automatically quantify subnuclear proteins dynamics in a time-resolved manner in living cells.

### Computational segmentation of nuclei and nucleoli using the machine learning-based tools Ilastik and StarDist

**Figure 2.**
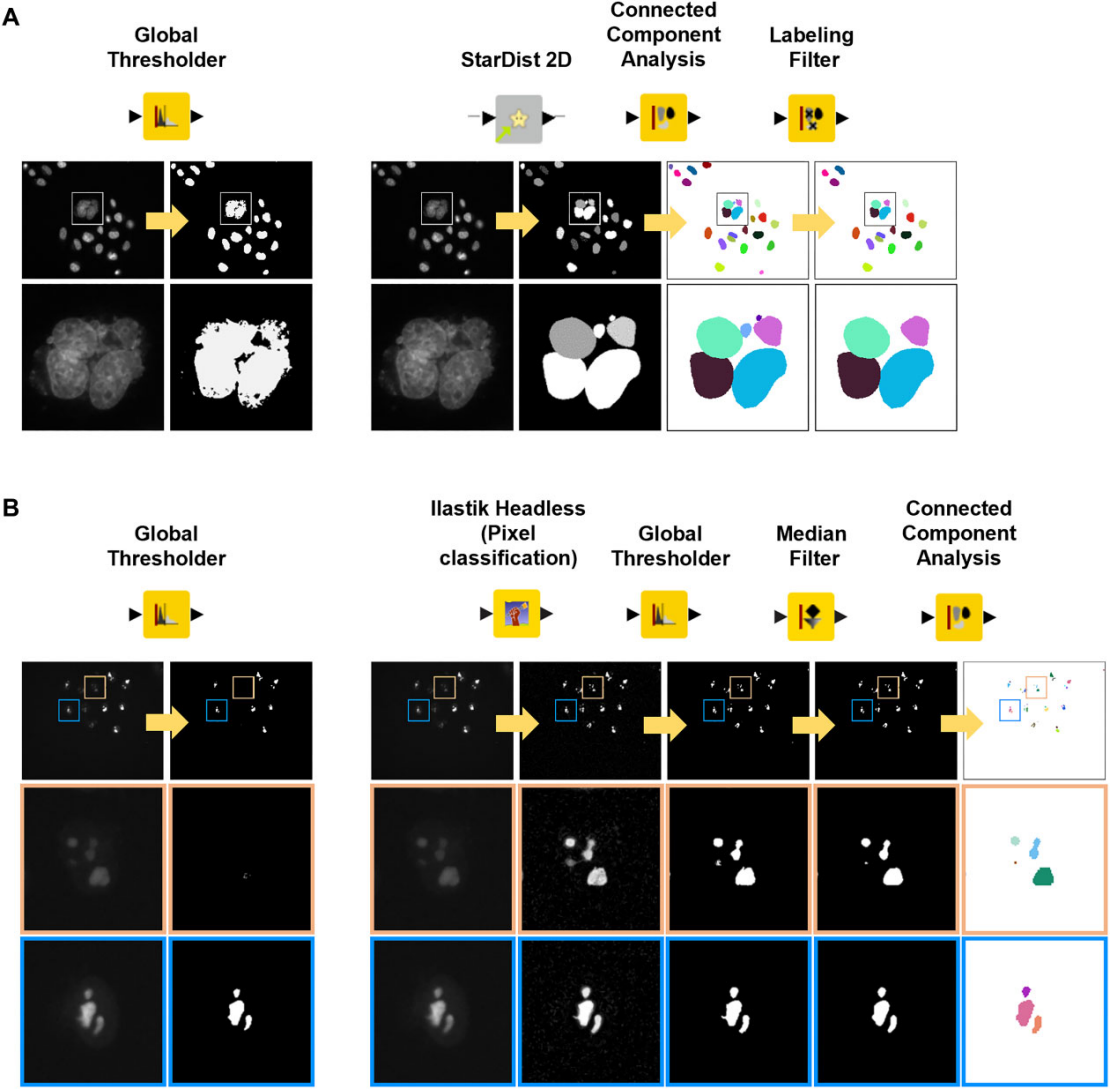
Image analysis strategies for the segmentation of nuclei and nucleoli. (**A**) Nuclei segmentation using the ‘*Global Thresholder’* (left panel) or the machine learning-based nuclei segmentation tool StarDist (right panel). The ‘*Connected Component Analysis’* node was used for instance segmentation (i.e. detection of individual nuclei). The ‘*Labeling Filter’* node removes objects, which are either too small or in contact with the edge of the image. Frames indicate magnified image regions. (**B**) Nucleoli segmentation using ‘*The Global Thresholder’* node (left panel) or the machine learning-based tool Ilastik (right panel). The ‘*Ilastik Headless (Pixel Classification)’* node produces probability maps (ranging from 0 to 1), which are transformed to binary images using the ‘*Global Thresholder’* node (default: Otsu). The *Median Filter* is applied to smooth the edges. Finally, the ‘*Connected Component Analysis’* node is used for detection of individual nucleoli. Blue frames indicate nucleoli with high NPM1-mCherry signals, while orange frames highlight nucleoli with low NPM1-mCherry intensities.

Image segmentation is the process of partitioning an image into various subgroups called segments [reviewed in (45)]. The simplest and fastest image segmentation technique is image thresholding, which partitions an image into foreground and background, thereby converting a greyscale image into a binary image. This is typically accomplished by either using a single threshold value for the entire image (‘global thresholding’) or several different threshold values, which are dynamically calculated for defined regions of the image to adapt to, e.g. varying illumination throughout the image (‘local adaptive thresholding’). The applicability of traditional, thresholding-based methods for nuclei detection is significantly limited when spatial configurations occur, such as cell clustering or cells in direct contact with each other. Therefore, advanced nuclei segmentation methods, including deep learning approaches, have been developed by several groups and whole consortia [reviewed in (46)]. In the approach of this study, we implemented such techniques into the above described KNIME workflow in order to enhance the robustness of the method. We first tested the ‘*Global Thresholder’* node in KNIME, which implements different thresholding algorithms, including the well-known *Otsu's* method (47), for its performance in nuclei segmentation (Figure 2A, left panel). When using this method, it was not possible to reliably distinguish between single cells in the presence of cell clusters. Using the deep learning tool StarDist, which is particularly useful when segmenting star-like or elongated objects, such as cell nuclei or neurons (38), significantly improved the nuclei segmentation (Figure 2A, right panel). To assign a unique label to each nucleus the ‘*Connected Component Analysis’* node was applied subsequently. Finally, the ‘*Labeling Filter’* node was incorporated into the workflow to remove objects that are too small, such as cell debris or air bubbles, as well as objects touching or converging with the edge of the image.

Next, we tested the suitability of the ‘*Global Thresholder’* node (default: Otsu) for nucleoli segmentation. While this thresholding method led to sufficient segmentation results when cells showed high NPM1-mCherry expression levels (Figure 2B, left panel, blue frames), low NPM1-mCherry intensities could not be detected (*cf*., orange frames). Therefore, for the segmentation of nucleoli, the ‘*Ilastik Headless’ (‘Pixel Classification’)* node was implemented into the workflow (Figure 2B, right panel). Ilastik is a ready-to-use, interactive, machine learning-based tool, which provides pre-defined workflows for image segmentation, object classification, counting or tracking (39). It uses the concept of supervised machine learning in which an algorithm (‘the classifier’) is trained by the user on input data to create a model that makes predictions. Once the classifier is sufficiently trained, the generated model can be applied to new data without supervision. Here, the pixel classification workflow, which produces semantic segmentations of images, was used. To train the classifier the two classes ‘*nucleolus*’ and ‘*background*’ were defined and examples for each class were provided by ‘clicks’ and ‘brush strokes’. Using Ilastik, nucleoli with low as well as high NPM1-mCherry signals were reliably detected. Since the ‘*Ilastik Headless’* node produces probability maps ranging from 0 to 1, the ‘*Global Thresholder’* node was included downstream to convert the probability images into binary images. Subsequently, the ‘*Median Filter’* node was included to smooth the edges, followed by the ‘*Connected Component Analysis’* node, which performed instance segmentation and resulted in the assignment of a unique labeling to each nucleolus. While executing the segmentation procedures, we initially experienced the occurrence of a Java heap space error, which is one of the most common issues when it comes to Java heap memory handling. This could be fixed by increasing KNIME’s memory usage limit and by implementing the ‘*Run Heavy Garbage Collector’* node in both segmentation meta nodes to achieve a memory reclaim.

In summary, by integrating the machine learning-based tools StarDist and Ilastik into the KNIME workflow, nuclei and nucleoli were successfully segmented in live-cell imaging data, which are critical steps in the automated analysis of subnuclear protein localizations.

### H_2_O_2_-induced nucleolar-nucleoplasmic protein shuttling in cells transiently transfected with eGFP-tagged wildtype PARP1

**Figure 3.**
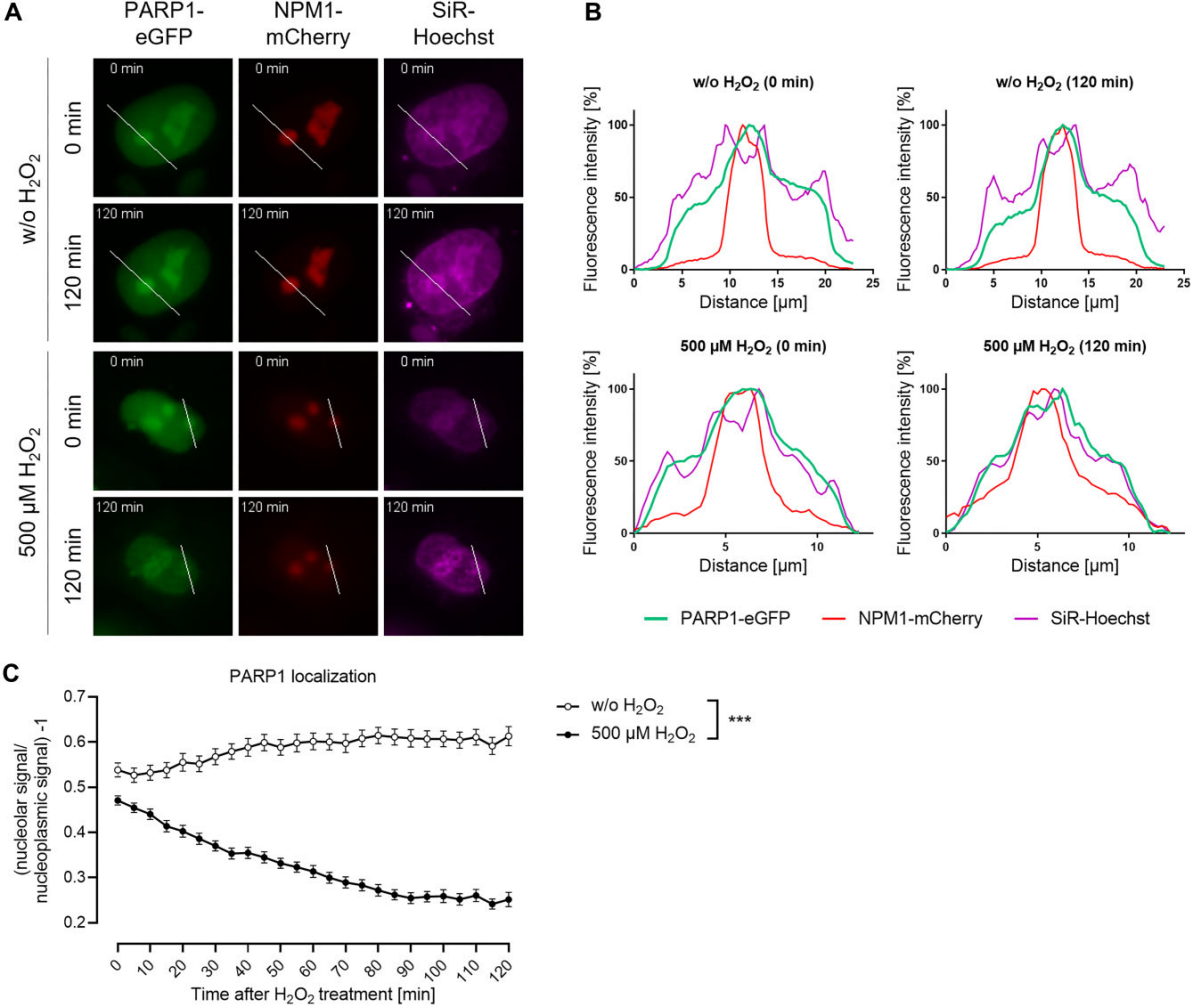
H_2_O_2_-induced translocation of PARP1 from nucleoli to the nucleoplasm in transiently transfected cells. (**A**) After co-transfecting *PARP1* KO cells with eGFP-tagged PARP1 and NPM1-mCherry, cells were mock-treated with DMEM or were treated with 500 μM H_2_O_2_. Immediately after treatment, live-cell imaging was performed for 2 h in 5 min intervals. To visualize nuclei, cells were incubated with SiR-Hoechst 3 h before live-cell imaging. For better visibility linear adjustments of brightness and contrast were performed using Fiji. (**B**) Intensity profiles were generated and normalized as described in the Material and Methods section. (**C**) Quantitative analysis by the newly developed KNIME workflow of the data shown in A. Data represent means ± SEM of 15 microscopic images per experiment typically containing >20 cells per image of three independent experiments.

To study H_2_O_2_-induced nucleolar-nucleoplasmic PARP1 shuttling in living cells we co-transfected HeLa *PARP1* KO cells, which have been previously generated and comprehensively characterized (18), with eGFP-tagged PARP1 and NPM1-mCherry (Figure 3A). For visualization of nuclei, we added 50 nM of the live-cell nuclei stain SiR-Hoechst at least 3 h before image acquisition. To induce genotoxic stress, cells were treated with 500 μM of the oxidizing agent H_2_O_2_ and live-cell image acquisition was started immediately thereafter. To assess the subnuclear localization of PARP1 and validate the newly developed KNIME workflow for image analysis, we employed both manual image analysis in Fiji (Figure 3B) and the newly developed KNIME workflow (Figure 3C). Intensity profiles obtained from the manual image analysis showed a clear co-localization of PARP1 with NPM1 in untreated cells, which is in agreement with a previous study showing an interaction of PARP1 with NPM1 (24). After 120 min of H_2_O_2_ treatment, a large proportion of PARP1 was released from the nucleoli. [*N.B.*, while we cannot fully exclude that the decrease in nucleolar PARP1 signal intensity is due to PARP1 degradation, we consider this option rather unlikely, since qualitative visual image analysis indicated the release of PARP1 from nucleoli to the nucleolar periphery (see, e.g. Figure 5B, Supplementary Figure S3, or Supplementary Video 6)]. Intriguingly, the intensity profiles revealed a strong co-localization of PARP1 with SiR-Hoechst 120 min after H_2_O_2_ treatment, which was especially pronounced in regions surrounding the nucleoli, most probably representing the peri-nucleolar heterochromatin. While it has been reported that NPM1 partially can relocate into the nucleoplasm upon H_2_O_2_ treatment (32), importantly, in our setting the subnuclear localization of NPM1-mCherry was not significantly affected (Figure 3A and B). This confirmed that NPM1 represents a suitable marker for nucleolar region determination under the conditions applied in our setting. It is important to note that even if a small portion of NPM1 is released from nucleoli following H_2_O_2_ treatment, this is not anticipated to impact the quantitative analysis of the protein of interest. This is because NPM1 signals solely functioned as a qualitative marker to identify nucleolar regions and were not subjected to quantitative analysis. Furthermore, the maintained subnucleolar localization of NPM1 indicates that overall nucleolar structure remained stable during the observation period and nucleoli did not disintegrate. The automated image analysis via KNIME allowed monitoring of the subnuclear localization of PARP1 over time and showed a significant relocalization of PARP1, which started immediately after H_2_O_2_ treatment and reached a plateau at around 90 min after the treatment. Taken together, using the newly developed KNIME workflow, we were able to monitor H_2_O_2_-induced nucleolar-nucleoplasmic shuttling of PARP1 in a time-resolved manner in living cells. Interestingly, our manual analysis in Fiji revealed that after H_2_O_2_ treatment, PARP1 accumulates at the nucleolar periphery.

### H_2_O_2_-induced nucleolar-nucleoplasmic protein shuttling in cells transiently transfected with eGFP-tagged TARG1 or APE1

**Figure 4.**
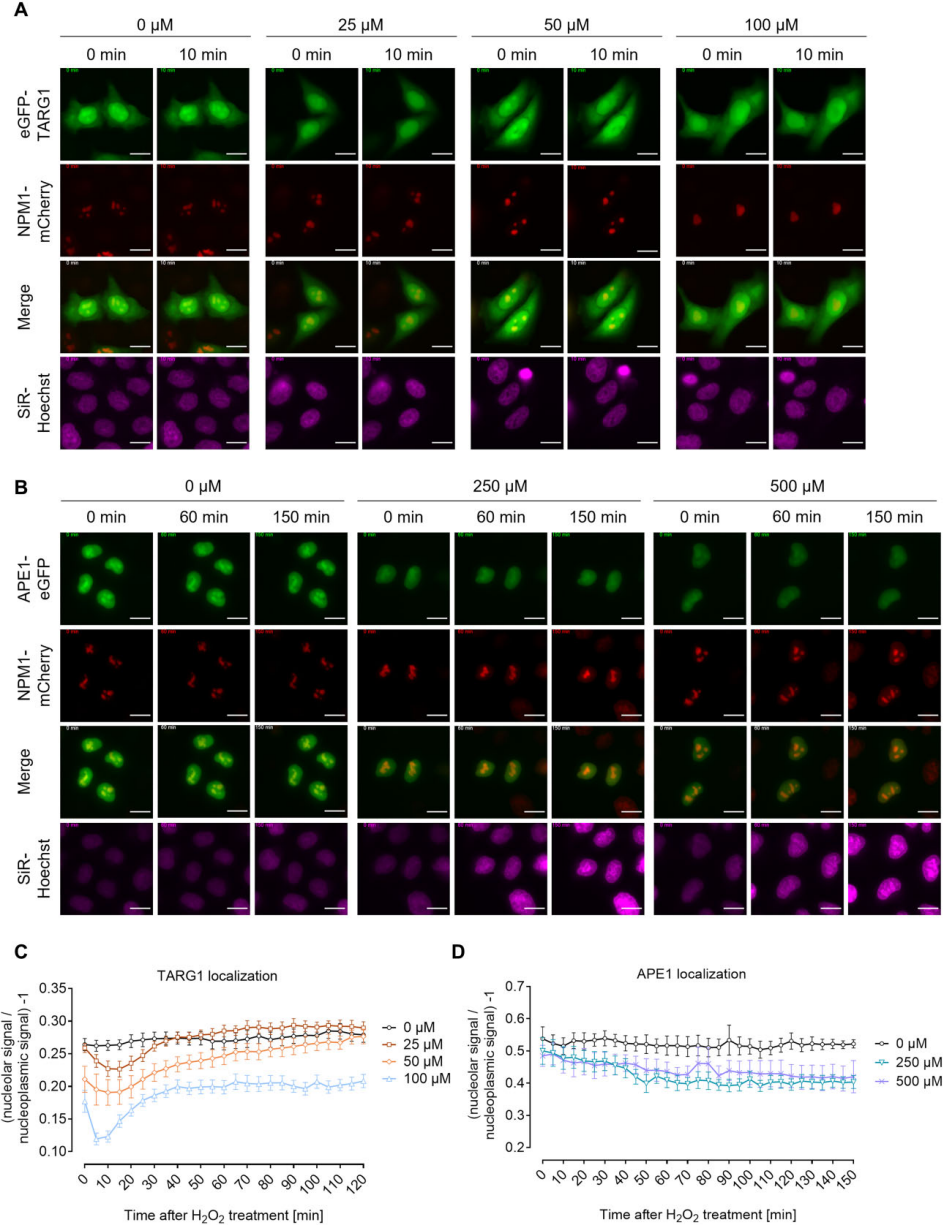
TARG1 and APE1 localization dynamics upon H_2_O_2_ treatment. For visualization of nuclei, cells were stained with SiR-Hoechst 3 h prior to live-cell imaging. Oxidative stress was induced using different concentrations of H_2_O_2_ as indicated. (**A**) Cells were transiently co-transfected with eGFP-TARG1 and NPM1-mCherry. Representative images at the time points 0 and 10 min are shown. (**B**) Representative images of cells co-transfected with APE1-eGFP and NPM1-mCherry at the time points 0, 60 and 150 min are shown. (A, B) For better visibility linear adjustments of brightness and contrast were performed using Fiji. Scale bars represent 20 μm. Merged images are of eGFP and mCherry channels. (**C**) Quantitative automated image analysis via KNIME of microscopic data shown in A. Data represent means ± SEM of five microscopic images typically containing >20 cells each of one proof-of-principle experiment. (**D**) Quantitative automated image analysis via KNIME of microscopic data shown in B. Data represent means ± SEM of four microscopic images typically containing >20 cells of one proof-of-principle experiment.

As a proof of concept, we next studied other nucleolar proteins, known to undergo dynamic shuttling upon genotoxic stress, including the macrodomain-containing terminal ADP-ribose protein glycohydrolase 1 (TARG1) and the BER protein apurinic/apyrimidinic endonuclease 1 (APE1). TARG1 was previously shown to mainly localize to transcriptionally active nucleoli through binding to rRNA, ribosomal proteins, as well as proteins associated with rRNA proteins and ribosomal assembly factors (48). Also, in response to H_2_O_2_-induced DNA damage, TARG1 undergoes nucleolar-nucleoplasmic shuttling in a PAR-dependent manner. To test, whether this H_2_O_2_-induced release of TARG1 can also be monitored using our newly developed live-cell imaging platform, we co-transfected HeLa WT cells with an eGFP-tagged TARG1 construct and NPM1-mCherry and treated cells with different concentrations of H_2_O_2_. Consistent with the previous data (48), results obtained with the new analysis pipeline revealed a fast release of TARG1 from nucleoli into the nucleoplasm after H_2_O_2_ treatment, which occurred in a concentration-dependent manner (Figure 4A,B, Supplementary Videos 1 and 2).

Next, we transfected HeLa WT cells with an eGFP-tagged APE1 construct and NPM1-mCherry. APE1 is predominately localized to nucleoli, where it interacts with NPM1, and was previously shown to translocate to the nucleoplasm and cytoplasm upon treatment with different agents, such as actinomycin D or cisplatin (36,49). To test whether treatment with H_2_O_2_ also results in a translocation of APE1, we treated cells with H_2_O_2_ and performed live-cell imaging over a time period of 3 h. In contrast to TARG1, there was only a slight release of APE1 from nucleoli into the nucleoplasm after H_2_O_2_ treatment_,_ suggesting that nucleolar-nucleoplasmic shuttling of APE1 occurs in a toxicant-specific manner (Figure 4C, D, Supplementary Videos 3 and 4).

In summary, these experiments proved the high-content live-cell imaging platform as a robust, sensitive, and versatile tool to analyze genotoxic stress-induced nucleolar-nucleoplasmic shuttling of different nucleolar proteins in living cells.

### H_2_O_2_-induced nucleolar-nucleoplasmic PARP1 shuttling in cells stably expressing PARP1-eGFP and NPM1-mCherry

**Figure 5.**
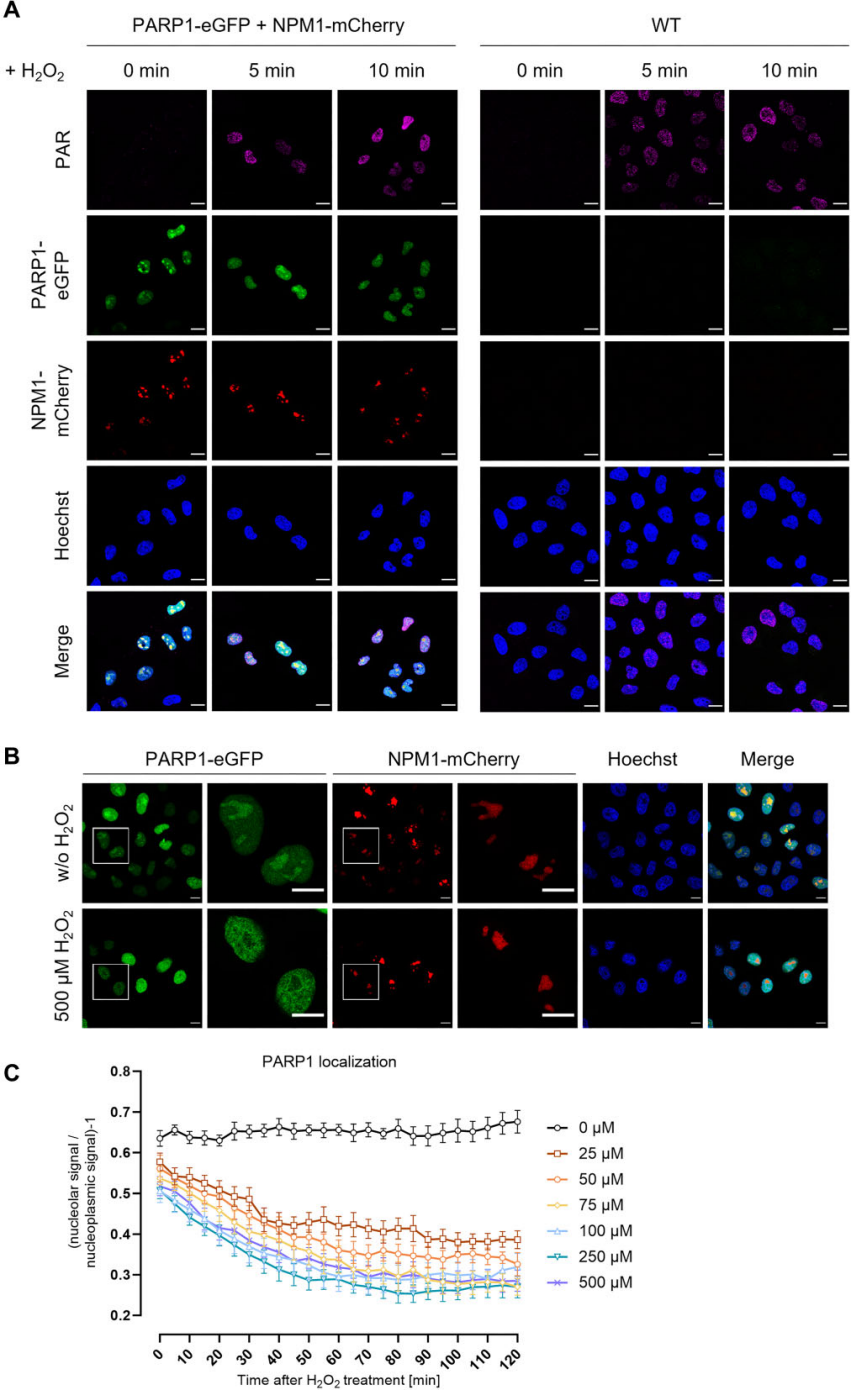
HeLa ‘PARP1-eGFP + NPM1-mCherry’ cell line displays intact PARylation activity and shows nucleolar-nucleoplasmic shuttling of PARP1 upon H_2_O_2_ treatment. (**A**) Representative images from confocal microscopy are shown. HeLa WT cells were included in the experimental setup as a positive control. Cells were mock-treated with DMEM or were treated with 500 μM H_2_O_2_ for 5 or 10 min. Samples were subjected to immunofluorescence staining for PAR. The Hoechst stain was used to visualize nuclei. Scale bars represent 20 μm. For quantification of PAR formation refer to Supplementary Figure S2A. (**B**) Cells were mock-treated with DMEM or were treated with 500 μM H_2_O_2_ for 2 h. Samples were subjected to Hoechst staining to visualize nuclei and analyzed by confocal microscopy. White frames indicate magnified image regions. Scale bars represent 20 μm. For better visibility linear adjustments of brightness and contrast were performed using Fiji. (**C**) Quantitative automated analysis of live-cell imaging data. For representative microscopic images refer to Supplementary Figure S3. Cells were treated with different concentrations of H_2_O_2_ as indicated, images were acquired using the live-cell imaging microscopic system Celldiscoverer 7 and analyzed via the KNIME workflow as described in the Material and Methods section. Data represent means ± SEM of five microscopic images typically containing >20 cells per image of a representative experiment.

Commonly used liposomal transfection reagents can activate stress response pathways in cells (50). To reduce cellular stress, to enhance sample throughput for pharmacological testing of compounds, and to further increase the sensitivity of the method, we generated a HeLa reporter cell line stably expressing PARP1-eGFP and NPM1-mCherry in a *PARP1* KO genetic background. To test whether the cell line shows normal PARylation activity upon induction of genotoxic stress, HeLa ‘PARP1-eGFP + NPM1-mCherry’ cells or HeLa WT cells were treated with 500 μM H_2_O_2_ followed by immunofluorescence staining with an anti-PAR antibody to analyze the PARylation response. Confocal microscopy revealed strong PARylation in the stable cell line 5 and 10 min after the treatment that was elevated in transgenic cells compared to WT cells (Figure 5A and Supplementary Figure S2A). We next treated the cells for 2 h with 500 μM H_2_O_2_ to see, whether H_2_O_2_ induces nucleolar-nucleoplasmic shuttling of PARP1-eGFP in this cell line. Using confocal microscopy, we could detect a strong release of PARP1 from nucleoli into the nucleoplasm in cells 2 h after H_2_O_2_ treatment, but not in cells mock-treated with medium only (Figure 5B). To further characterize protein expression in the reporter cell line, we performed Western blot experiments, analyzing PARP1 and NPM1 expression in both HeLa WT and transgenic HeLa ‘PARP1-eGFP + NPM1-mCherry’ cells (+/– H_2_O_2_ treatment), followed by densitometric analysis. Our results indicate that, as expected, PARP1-eGFP levels in the transgenic cell line were approximately four times higher compared to WT cells (Supplementary Figure S2B and S2C). Importantly, PARP1 levels remained constant following H_2_O_2_ treatment in both WT and transgenic cells (Supplementary Figure S2B and S2C). Furthermore, quantification of mean fluorescence intensities of nucleoplasmic regions and the nucleoli confirmed that the PARP1 signal declined in nucleoli, while remaining constant in the nucleoplasm (Supplementary Figure S3B). Similarly, analysis of endogenous NPM1 levels revealed stability upon H_2_O_2_ treatment in both WT and transgenic cells. Additionally, NPM1-mCherry expression was detectable in the transgenic cell line (Supplementary Figures S2B and S2C).

We next performed a live-cell imaging experiment, in which the HeLa reporter cell line was treated with concentrations of H_2_O_2_ ranging from 25 to 500 μM to test the sensitivity of the method (representative images are shown in Supplementary Figure S3, for representative videos see Supplementary Videos 5 and 6). Data obtained with the automated KNIME analysis revealed a nucleolar release of PARP1 after treatment with H_2_O_2_ at a concentration as low as 25 μM (Figure 5C). Higher H_2_O_2_ concentrations showed a trend towards an even more prominent PARP1 release. Based on these data, we continued with 50 μM H_2_O_2_ in the following experiments in which we tested the effect of a range of pharmacological substances on the nucleolar-nucleoplasmic PARP1 shuttling. Taken together, the HeLa ‘PARP1-eGFP + NPM1-mCherry’ reporter cell line can be used for mechanistic and screening studies on nucleolar-nucleoplasmic protein translocations. Using this cell line, it was possible to detect cellular effects of H_2_O_2_ treatment at concentrations as low as 25 μM, which is thought to be close to physiological extracellular concentrations [reviewed in (51)].

### Screening of small molecule inhibitors for effects on nucleolar-nucleoplasmic PARP1 shuttling

**Figure 6.**
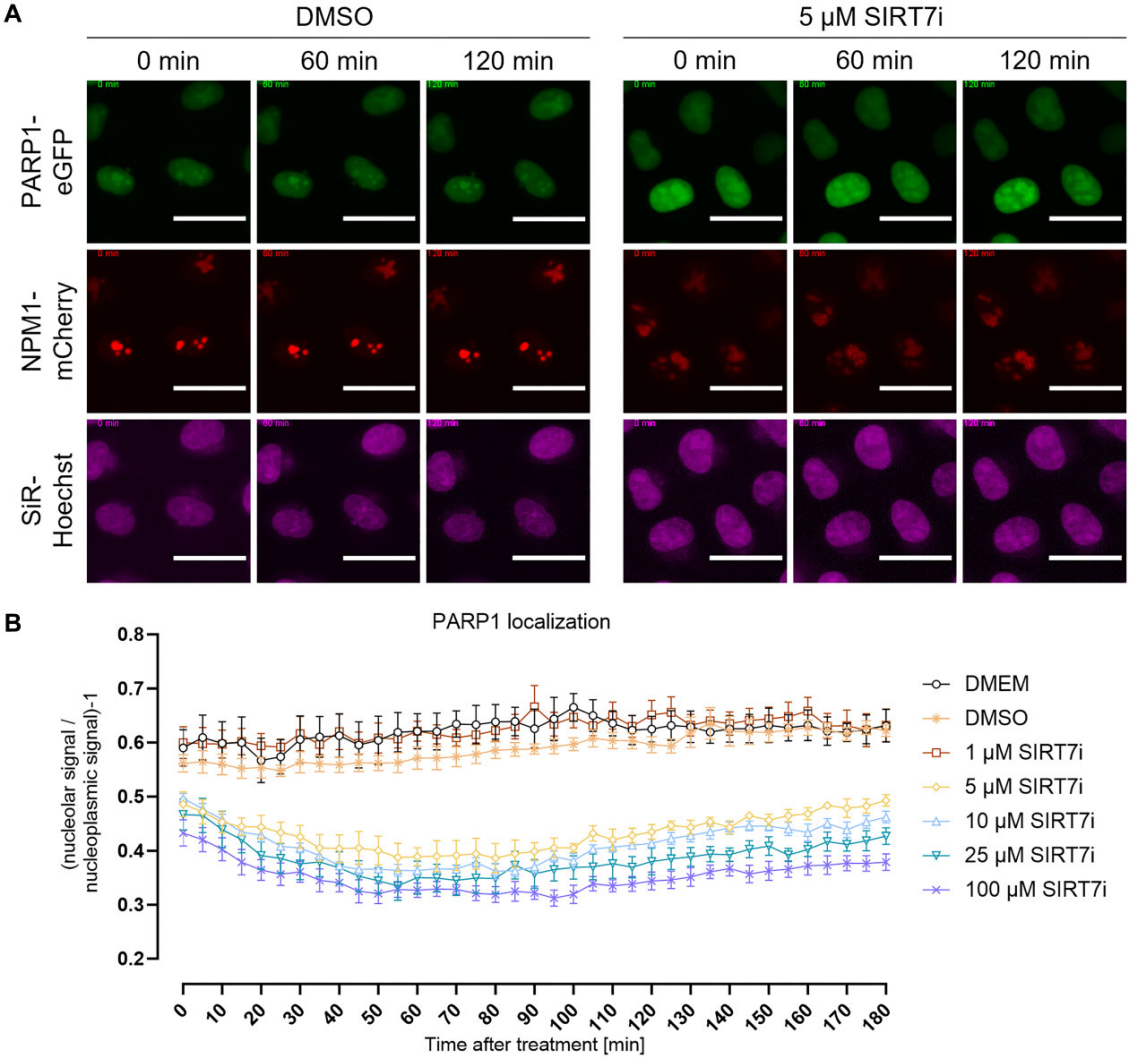
SIRT7 inhibition leads to release of PARP1 from nucleoli. For visualization of nuclei, cells stably expressing PARP1-eGFP and NPM1-mCherry were stained with SiR-Hoechst 3 h prior to live-cell imaging. (**A**) Cells were pre-incubated with different concentrations of the SIRT7 inhibitor 97 491 for 30 min, then live-cell imaging was performed for 180 min in 5 min intervals. Representative magnified images of cells treated with DMSO (solvent control) or 5 μM of the SIRT7 inhibitor 97 491 are shown. For better visibility linear adjustments of brightness and contrast were performed using Fiji. Scale bars represent 20 μm. (**B**) Quantitative automated image analysis via KNIME of data shown in A. Data represent means ± SEM of five microscopic images typically containing >20 cells per image of one proof-of-principle experiment.

After validating the ‘PARP1-eGFP + NPM1-mCherry’ reporter cell line, we performed a pharmacological screen with different inhibitors of enzymes catalyzing post-translational modifications to identify possible regulators of the H_2_O_2_-induced nucleolar release of PARP1. Several of the tested inhibitors are currently in clinical development. The spectrum of inhibitors included the ATM inhibitor AZD0156, the ATR inhibitor ceralasertib, the MRN (Mre11, Rad50 and Nbs1) complex inhibitor (Z)-mirin, the sirtuin 7 (SIRT7) inhibitor 97 491 as well as staurosporine, a well-known broad spectrum kinase inhibitor. To determine suitable working concentrations and to analyze the effect of the inhibitors on the subnuclear localization of PARP1, cells were first treated with various concentrations of the inhibitor compounds only, i.e. without H_2_O_2_ treatment. To analyze the effect of the inhibitors on H_2_O_2_-induced nucleolar-nucleoplasmic PARP1 shuttling, afterwards co-treatments with H_2_O_2_ were performed. As it is evident from Supplementary Figure S4 AZD0156, ceralasertib, (Z)-mirin, and staurosporine did not significantly affect the nucleolar-nucleoplasmic shuttling of PARP1, neither alone nor in combination with H_2_O_2_ treatment. Intriguingly, however, treatment with the SIRT7 inhibitor 97 491 alone resulted in a loss of nucleolar PARP1 accumulation starting at a concentration of 5 μM, suggesting a role for SIRT7 activity in nucleolar homeostasis (Figure 6, Supplementary Figure S5). In summary, these experiments verified the suitability of the system to analyze pharmacological effects on nucleolar-nucleoplasmic protein shuttling in an automated manner, with a significantly higher throughput and data density compared to previously used methods based on non-live cell imaging techniques. Furthermore, these data point to a role of SIRT7 in regulating PARP1’s subnuclear localization, which opens avenues for future in-depth mechanistic studies.

### Effect of pharmacological PARP inhibition on H_2_O_2_-induced nucleolar-nucleoplasmic PARP1 shuttling

Since none of the inhibitors mentioned above significantly affected the H_2_O_2_-induced nucleolar-nucleoplasmic shuttling of PARP1, we analyzed to what extent PARP activity itself may contribute to the shuttling effect. PARPi have entered the clinic as chemosensitizers in combination with classical chemotherapeutics or as monotherapeutic agents in HR-deficient tumors following the concept of synthetic lethality [reviewed in (52)]. Here, we investigated the effect of different PARPi, including the four FDA-approved PARPi olaparib, rucaparib, niraparib and talazoparib on the H_2_O_2_-induced nucleolar-nucleoplasmic shuttling of PARP1. We pre-incubated HeLa ‘PARP1-eGFP + NPM1-mCherry’ cells with 1 μM or 10 μM of the various PARPi and then added 50 μM of H_2_O_2_ for 5 or 10 min. Then, cells were stained with the anti-PAR antibody 10H to detect PARylation and analyzed by confocal microscopy. As it is evident from Supplementary Figure S6, 1 μM of each inhibitor was sufficient to inhibit PARylation activity. To test whether the PARPi affect the subnuclear localization of PARP1 on their own, 1 μM or 10 μM of the various PARPi were added to HeLa ‘PARP1-eGFP + NPM1-mCherry’ cells and live-cell imaging was started immediately thereafter (Supplementary Figure S7). The automated analysis of the imaging data via KNIME revealed a moderate release of PARP1 shortly after starting the image acquisition in all tested conditions, including the controls (Supplementary Figure S7B). This release was transient in most of the conditions tested, except for 10 μM veliparib and 10 μM rucaparib, which led to a longer retention of PARP1 in the nucleoplasm, potentially by inducing low-level genotoxic stress.

**Figure 7.**
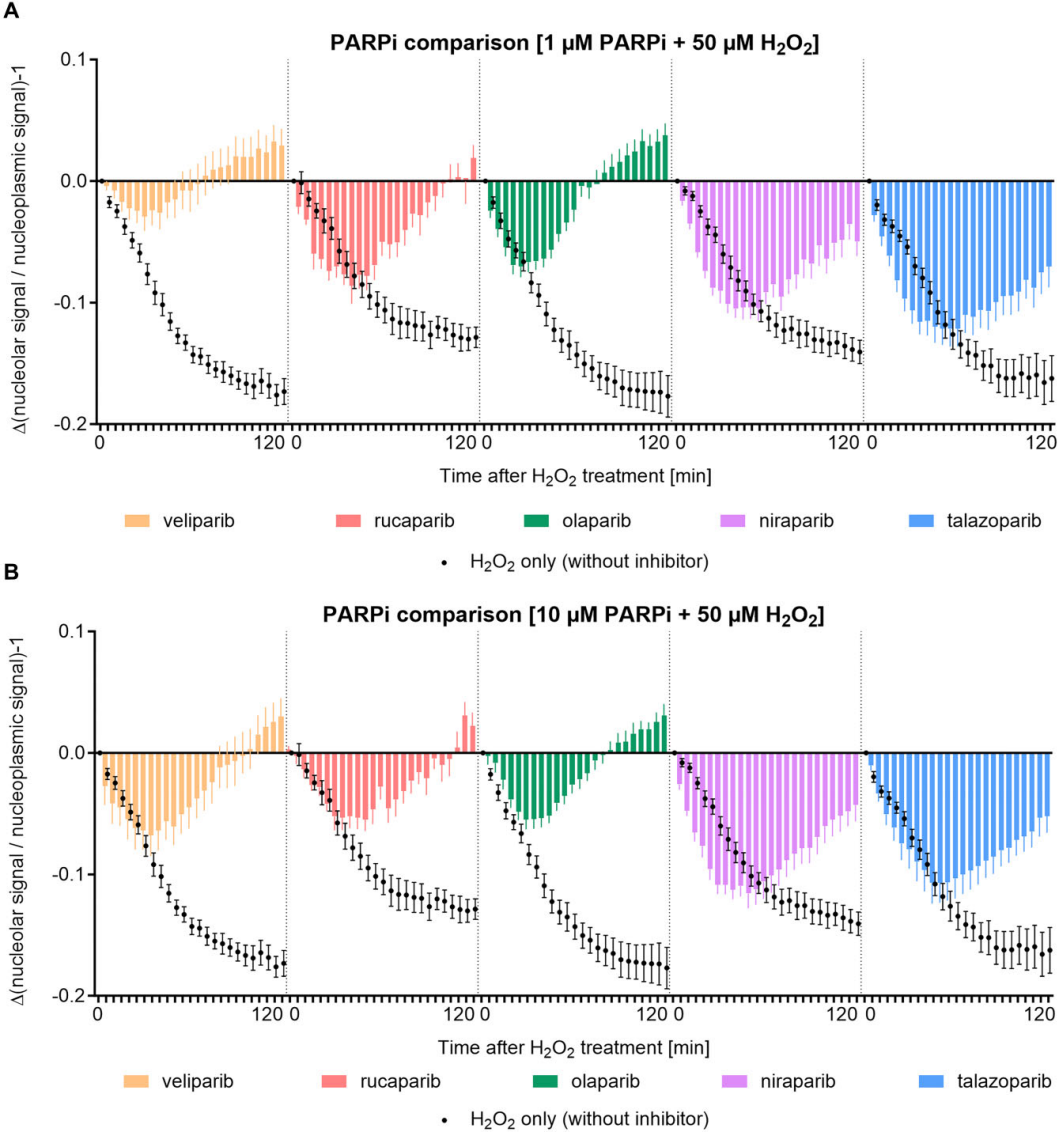
PARP1 localization upon co-treatment with pharmacological PARP1 inhibitors and H_2_O_2_. HeLa ‘PARP1-eGFP + NPM1-mCherry’ cells were pre-incubated with 1 μM (**A**) or 10 μM (**B**) of veliparib, rucaparib, olaparib, niraparib or talazoparib for 30 min. Afterwards, 50 μM of H_2_O_2_ were added to the cells to induce oxidative stress. Immediately after the H_2_O_2_ treatment, live-cell imaging was performed for 120 min every 5 min. Depicted are changes in the ratios of nucleolar to nucleoplasmic PARP1 signal as evaluated via the KNIME workflow. Data sets with full circles (•) represent corresponding H_2_O_2_-treated only samples, respectively. Experiments for both concentrations of each inhibitor, including H_2_O_2_-only treated samples, were conducted on a single 96-well plate. Consequently, the datasets of H_2_O_2_-only treated samples are identical between panels A and B. For full data sets including negative controls refer to Supplementary Figure S9. Data represent means ± SEM of five microscopic images per experiment typically containing >20 cells per image of three independent experiments.

Next, we tested whether PARP inhibition affects the H_2_O_2_-induced nucleolar-nucleoplasmic shuttling of PARP1. We therefore pre-incubated ‘PARP1-eGFP + NPM1-mCherry’ cells with various PARPi for 30 min and then challenged cells with 50 μM H_2_O_2_. Immediately after the H_2_O_2_ treatment, live-cell imaging was performed and the acquired imaging data (Supplementary Figure S8) were analyzed using the KNIME workflow. For better comparability, the absolute changes in nucleolar to nucleoplasmic PARP1 signal ratios were calculated and plotted against time (Figure 7). The graphs showing the pre-processed data are depicted in Supplementary Figure S9. Interestingly, the inhibitors affected the nucleolar-nucleoplasmic shuttling of PARP1 with considerable variability. Veliparib was the only inhibitor that strongly inhibited H_2_O_2_-induced nucleolar release of PARP1 at a concentration of 1 μM. Pre-incubation with 1 μM rucaparib or olaparib, followed by H_2_O_2_ treatment resulted in a nucleolar release of PARP1 nearly to the same extent as after treatment with H_2_O_2_ alone, but 60 to 95 min after H_2_O_2_ treatment PARP1 was completely relocated to nucleoli. Niraparib and talazoparib, which is known to be very potent in trapping PARP1, led to a slightly faster nucleolar release of PARP1 upon H_2_O_2_ treatment and a partial nucleoplasmic retention of PARP1 as compared to the other inhibitors.

To test if inhibition of PAR degradation affects nucleolar-nucleoplasmic PARP1 shuttling, we employed the potent, cell permeable PARG inhibitor PDD00017273. Treatment with different concentrations of the inhibitor alone as well as co-treatment with H_2_O_2_ showed no effect on the subnuclear PARP1 localization or H_2_O_2_-induced nucleolar-nucleoplasmic PARP1 shuttling (Supplementary Figures S10 and S11).

In summary, these experiments revealed that PARPi modify H_2_O_2_-induced nucleolar-nucleoplasmic shuttling of PARP1 in a compound-specific manner, pointing to subtle differences in the mechanisms of action of the different PARPi.

### H_2_O_2_-induced increase in SiR-Hoechst intensity

Interestingly, throughout this study we observed an increase in SiR-Hoechst intensity upon H_2_O_2_ treatment. It is known that upon DNA damage induction, PARP1 and its enzymatic activity play a crucial role in chromatin relaxation (19). Therefore, we hypothesized that the observed increase in SiR-Hoechst staining may be due to H_2_O_2_-induced chromatin relaxation, enhancing the dye's accessibility to DNA. To test whether PARP inhibition can counteract the H_2_O_2_-induced increase in SiR-Hoechst intensity, we analyzed the data shown in Supplementary Figure S12 via KNIME with respect to SiR-Hoechst intensity. As it is evident from Supplementary Figure S12, treatment with 1 μM of each of the PARPi tested, was sufficient to prevent the H_2_O_2_-induced increase in SiR-Hoechst intensity.

In summary, these data indicate that PARP1-mediated PAR formation is at large responsible for the H_2_O_2_-induced increase in SiR-Hoechst intensity. Importantly, there are no indications that this phenomenon affects the specificity, sensitivity and validity of the method presented herein, since SiR-Hoechst intensities were not quantitatively evaluated during the image segmentation procedures.

## Discussion

Recent advancements in live-cell imaging techniques enable the examination of dynamic subcellular processes in real-time. Also, significant progress has recently been achieved in terms of cell tracking and segmentation (53). In the DNA repair field, live-cell imaging was extensively used to study the spatio-temporal recruitment of DNA repair factors to laser-induced DNA lesions [reviewed in (54)]. Moreover, advanced imaging techniques, such as fluorescence recovery after photobleaching (FRAP), were successfully applied to assess the turnover of DNA repair proteins at DNA damage sites (25,55–57). Despite the great advances in live-cell microscopy, our understanding of genotoxic stress-induced nucleolar-nucleoplasmic protein shuttling is mainly derived from imaging studies in fixed cells, which can only provide snapshots of the rapid protein dynamics. Possible reasons for this are that live-cell imaging is challenging in terms of maintaining cells in a ‘healthy’ state, while performing the imaging procedure, and that handling and analyzing the significant amounts of acquired imaging data during live-cell imaging are demanding in many aspects.

In the present work, we combined high-content live-cell microscopy with a newly developed automated KNIME workflow to investigate stress-induced nucleolar-nucleoplasmic protein shuttling in living cells. Compared to manual analyses, this approach is much less time-consuming and less prone to user-bias. Moreover, through the implementation of state-of-the-art tools, which are based on machine learning, this setup enables the analysis of even complex and ‘delicate’ data, e.g. in the presence of cell clusters.

Using this system, we were able to monitor the spatio-temporal dynamics of different transiently transfected eGFP-tagged DNA repair proteins, including PARP1, TARG1 and APE1, after treatment with the oxidizing agent H_2_O_2_. All of these proteins were previously described to undergo nucleolar-nucleoplasmic shuttling (26,36,48,49), thereby further validating the applicability of the newly developed method in analyzing stress-induced nucleolar-nucleoplasmic shuttling of various nucleolar proteins within living cells. Importantly, while in general potential phototoxic effects can be an issue in fluorescence and in particular live-cell microscopy (58), we have no indications of such effects in our experimental setting. This is evident by the fact that no protein translocations were observed in non-treated cells and that even very low concentrations of H_2_O_2_ induced clearly visible effects on protein translocations suggesting only minor background damage. A potential caveat of the method arises from prior research by Yang et al., which demonstrated stress-induced NPM1 translocation at high doses of H_2_O_2_ (500 μM), while lower doses (125 μM) showed only minimal effects (32). In our study, at concentrations of up to 500 μM H_2_O_2_, we did not observe significant NPM1 relocalization. This suggests relatively mild stress conditions in our experimental setup, possibly attributed to the presence of scavenging components of FBS in the medium, which likely reduced effective H_2_O_2_ concentrations. Notably, we observed PARP1 relocalization after treatment with H_2_O_2_ concentrations as low as 25 μM, with pharmacological studies conducted at concentrations of 50 μM. These findings support the idea that nucleolar integrity and the bulk of nucleolar NPM1 localization were maintained under our treatment conditions. It's important to highlight that minor fluctuations in NPM1 signal intensities are not relevant to our methodology, as NPM1 signals primarily served as qualitative markers for nucleolar region determination. Quantification of NPM1 signal intensities was not performed at any stage.

In general, it is highly likely that swift nucleolar-nucleoplasmic shuttling of proteins is mediated by dynamic changes in PTMs, and examples of such mechanisms have been described before [(27–30) and reviewed in (23)]. Therefore, we set out to perform a pharmacological screen with inhibitors of enzymes that mediate PTMs, mainly in the context of DNA damage response, using the newly generated HeLa ‘PARP1-eGFP + NPM1-mCherry’ reporter cell line. While none of the tested inhibitors for ATM, ATR, MRE11, or the broad-spectrum kinase inhibitor staurosporine significantly affected the nucleolar-nucleoplasmic shuttling of PARP1, either alone or in combination treatment with H_2_O_2_, treatment with the SIRT7 inhibitor alone triggered a release of PARP1 from nucleoli, irrespective of co-treatment with H_2_O_2_. SIRT7 is one of seven members of the human sirtuin family [reviewed in (59)]. Sirtuins act as histone or protein deacetylases that use NAD^+^ as a substrate, thereby regulating a plethora of cellular processes, including cell metabolism, gene expression, apoptosis, and DNA repair. In addition, some other types of biochemical reactions, such as ADP-ribosylation, desuccinylation and demalonylation have been shown to be carried out by sirtuins [reviewed in (60,61)]. SIRT7 is the only sirtuin that is enriched in nucleoli (62). Recent studies revealed its involvement in multiple cellular processes, including ribosome biogenesis, tumorigenesis, stress response and genomic stability [reviewed in (63)]. In general, an interplay between PARPs and sirtuins has long been documented, since both families represent major consumers of cellular NAD^+^ [reviewed in (64)]. SIRT7 has been shown to directly interact with PARP1 in response to DNA damage (65) and several studies have found SIRT7 to be recruited to DSBs in a PARP1-dependent manner (65,66). Furthermore, SIRT7 is known to act as a positive regulator of ribosome biogenesis [reviewed in (67)]. Thus, knockdown or inhibition of SIRT7 catalytic activity have been demonstrated to result in decreased association of Pol I with rDNA and a reduction of RNA Pol I-driven transcription (68). The nucleolar localization of PARP1 on the other hand, has been shown to rely on active RNA Pol I transcription (24). Therefore, it is conceivable that upon SIRT7 inhibition, PARP1 is released from nucleoli due to downregulation of Pol I transcription.

Since none of the above-mentioned DNA damage response inhibitors significantly affected the nucleolar-nucleoplasmic PARP1 shuttling upon H_2_O_2_ treatment, we tested inhibitors of PARylation themselves for potential effects on PARP1 shuttling. Data from our previous study (26) already suggested that PARylation activity significantly influences nucleolar-nucleoplasmic protein shuttling of PARP1 and other DNA repair factors. Thus, detailed analysis of a selection of five different clinically relevant PARPi at two different concentration levels, i.e. 1 μM and 10 μM, revealed specific differences in H_2_O_2_-induced nucleolar-nucleoplasmic PARP1 shuttling (Figure 7 and Supplementary Figure S9). In general, co-treatment with H_2_O_2_ and PARPi resulted in a reduced retention of PARP1 in the nucleoplasm, compared to treatment with H_2_O_2_ only. This is somewhat counterintuitive, since PARPi are known to cause PARP1 persistency at damaged DNA sites. It is well possible that upon translocation of PARP1 to the nucleoplasm and catalytic activation at sites of DNA damage, PARP1 is unable to reenter the nucleoli due to auto-modification with PAR. In contrast, in case of inhibitor use, the unmodified PARP1 molecules may be able to do so, depending on their trapping properties at sites of DNA damage (see below).

It is intriguing to see that the five different PARP-specific inhibitors led to substantially different PARP1 shuttling dynamics after induction of oxidative stress by H_2_O_2_. It would be very interesting to unravel the underlying mechanisms of these effects to better understand the subtle, yet potentially clinically relevant, different mechanisms of action of these inhibitors.

One potential reason for the different effects may be caused by different potencies of the inhibitors. Interestingly, while at first glance all tested PARPi exhibit similar potencies with IC50 or *k*_i_ values in the low nanomolar range, some researchers in the field argue that this is a misconception [reviewed in (69)], since specific assays indeed were able to detect differences with talazoparib being significantly more potent than all other inhibitors, followed by rucaparib (70). This is corroborated by a recent study indicating that although the in-vitro IC50 values of talazoparib, olaparib, and veliparib were found to be comparable, their potency can vary by a factor of 1000-fold in cellular assays, with talazoparib demonstrating the highest potency and veliparib the lowest. (71). The observed effects on PARP1 shuttling dynamics after treatment with veliparib, oliparib and talazoparib may fit within this scheme of different PARPi potencies (i.e. veliparib and olaparib leading to reduced PARP1 retention in the nucleoplasm, and talazoparib leading to a stronger retention than veliparib and olaparib). Yet, obviously the effects of rucaparib and niraparib do not, since rucaparib is the second most potent inhibitor of the five and niraparib shows a potency comparable to the one of veliparib and olaparib (70).

Another potential cause for the observed differences in PARP1 shuttling dynamics may lie in the specific capacities of the PARPi to trap PARP1 at sites of DNA damage (72–74). While it has been proposed that inhibition of PARP1’s auto-PARylation, which normally promotes its dissociation from the DNA, drives PARP1 trapping (69,74,75), other researchers suggested that specific allosteric mechanisms contribute to PARP trapping (72). According to this view, PARPi do considerably vary in their PARP trapping potencies: talazoparib has been revealed to be by far the most potent at trapping PARP1, followed by niraparib, olaparib and rucaparib, which have approximately equal trapping potencies, and lastly veliparib, representing the weakest PARP trapper (76). A more recent study proposed that PARPi have diverse allosteric effects on PARP’s autoinhibitory helical domain (HD), which impact PARP1 binding to sites of DNA damage (77). According to this, PARPi can be classified into three types, with type II inhibitors promoting retention of PARP1 at damage sites due to an allosteric mechanism (i.e. talazoparib and olaparib), type III inhibitors resulting in an allosteric pro-release, thus acting as ‘anti-trappers’ (i.e. niraparib, rucaparib and veliparib), and finally type I PARPi exhibiting no allosteric effect at all (77).

Yet, the existence of such an allosteric mechanism could not be fully verified so far and is still under debate [reviewed in (69)]. Recently, the classical trapping mechanism was challenged by a study, which demonstrated a rapid exchange of PARP1 molecules even in the presence of PARPi by using quantitative live-cell imaging and FRAP (57). This finding was further supported by live-cell single molecule imaging experiments from another group, which revealed that most PARP1/2 molecules diffuse freely in nuclei regardless of DNA damage or the presence of PARPi. (78). The same study showed that talazoparib, but not olaparib, increased the number and retention time of a small fraction of chromatin-bound PARP1/2, demonstrating that trapping effects appear to exist, but seem to only affect a small proportion of PARP molecules. Our data are in line with these recent findings, by demonstrating that the majority of PARP1 molecules can freely relocate to nucleoli in the presence of PARPi, instead of being stalled at damaged DNA sites. At the same time, our observations correlate with the previously reported PARP trapping potencies (72) – except for niraparib, which is considered an ‘anti-trapper’, yet led to a similar nucleoplasmic retention of PARP1 as talazoparib.

Niraparib, however, may be a special case, as this inhibitor exhibits some unique binding properties [reviewed in (69)]. While all tested PARPi share three conserved interactions with the active sites of PARP1 and PARP2, each PARPi in addition forms unique interactions, which may in part explain differences in their affinities [reviewed in (69)]. The least potent PARPi niraparib has been reported to build two unique interactions with the HD, which represents a highly conserved subdomain in DNA-dependent PARPs (i.e. PARP1-3). These unique binding interactions have been proposed to contribute to the high selectivity of niraparib [(79) and reviewed in (69)].

Taken together, it appears that several overlapping molecular mechanisms are contributing to the different effects observed in the current study.

Another interesting finding in this study was that after H_2_O_2_ treatment PARP1 accumulated at the nucleolar periphery, which is known to represent a site for heterochromatin localization [reviewed in (80)]. A previous study demonstrated that after each round of DNA replication PARP1 associates with TTF-1-interacting protein-5 (TIP5), which represents a subunit of the nucleolar remodeling complex (NoRC), via the noncoding pRNA and thereby mediates the inheritance of heterochromatic structures (81). Our data point towards an additional role of PARP1 in the PNH upon H_2_O_2_-induced genotoxic stress, which remains to be deciphered. To further clarify the molecular mechanisms driving H_2_O_2_-induced nucleolar-nucleoplasmic PARP1 shuttling, future studies could explore the effects of additional small molecules, such as inhibitors targeting NEDDylation, ubiquitination, or SUMOylation. Additionally, investigating the nuclear distribution patterns of established DNA damage markers, such as γ-H2A.X and 53BP1 foci, would provide valuable insights. While our current study focuses on H_2_O_2_-induced nucleolar-nucleoplasmic PARP1 shuttling, future research should also examine the dynamics of PARP1 and its underlying mechanisms in response to other genotoxic agents. Furthermore, exploring additional mechanistic aspects, such as the potential impact of rDNA and rRNA damage on rRNA transcription rates and turnover, presents an intriguing avenue for future investigation. Moreover, our newly developed method could be utilized to assess the effects of clinical PARPi on subnuclear PARP1 localization in cancer-relevant model systems, such as BRCA-deficient cells or cell lines with other homologous recombination deficiencies. Finally, by employing standardized data input and output formats, along with a wide selection of available plugins and extensions, which can be incorporated into the KNIME workflow, this method can be customized to suit the particular demands of different microscopic imaging platforms and experimental setups. Thus, while at the moment the method works with 2D imaging data, in principle, the method can be adapted for implementation in confocal or even super-resolution settings, thereby allowing the analysis of 3D imaging data. This may enable to study protein dynamics within the subnuclear space including the spatio-temporal behaviour of certain DNA repair foci. Thus, in general, the method's adaptability allows also for the exploration of inquiries extending beyond nucleolar-nucleoplasmic shuttling.

## Supplementary Material

gkae598_Supplemental_Files

## Data Availability

All relevant data are included in the main manuscript or the Supplementary Material file. Raw data are available upon reasonable request from the corresponding authors.
