# Supplementary material for: Monitoring nucleolar-nucleoplasmic protein shuttling in living cells by high-content microscopy and automated image analysis
Source: Nucleic Acids Res. 2024 Jul 22;52(15):e72. doi: 10.1093/nar/gkae598 (PMC11347172; doi:10.1093/nar/gkae598)
Supplement: gkae598_Supplemental_Files [file gkae598_supplemental_files.zip › Engbrecht et al_SI-R1-v3.pdf]

Supplementary information for

Monitoring nucleolar-nucleoplasmic protein shuttling in living cells by high-content microscopy and automated image analysis

Marina Engbrecht[1], David Grundei[1, †], Asisa M. Dilger[2, †], Hannah Wiedemann[1], Ann-Kristin Aust[1], Sarah Baumgärtner[1], Stefan Helfrich[3], Felix Kergl-Räpple[3], Alexander Bürkle[1], and Aswin Mangerich[1, 2] *

[1] Molecular Toxicology, Department of Biology, University of Konstanz, 78457, Konstanz, Germany

[2] Nutritional Toxicology, Institute of Nutritional Science, University of Potsdam, 14469 Potsdam, Germany

[3] KNIME GmbH, Reichenaustr. 11, 78467 Konstanz, Germany

[†] These authors contributed equally to this work

* Correspondence to: Aswin Mangerich, University of Potsdam, Germany (mangerich@uni-potsdam.de)

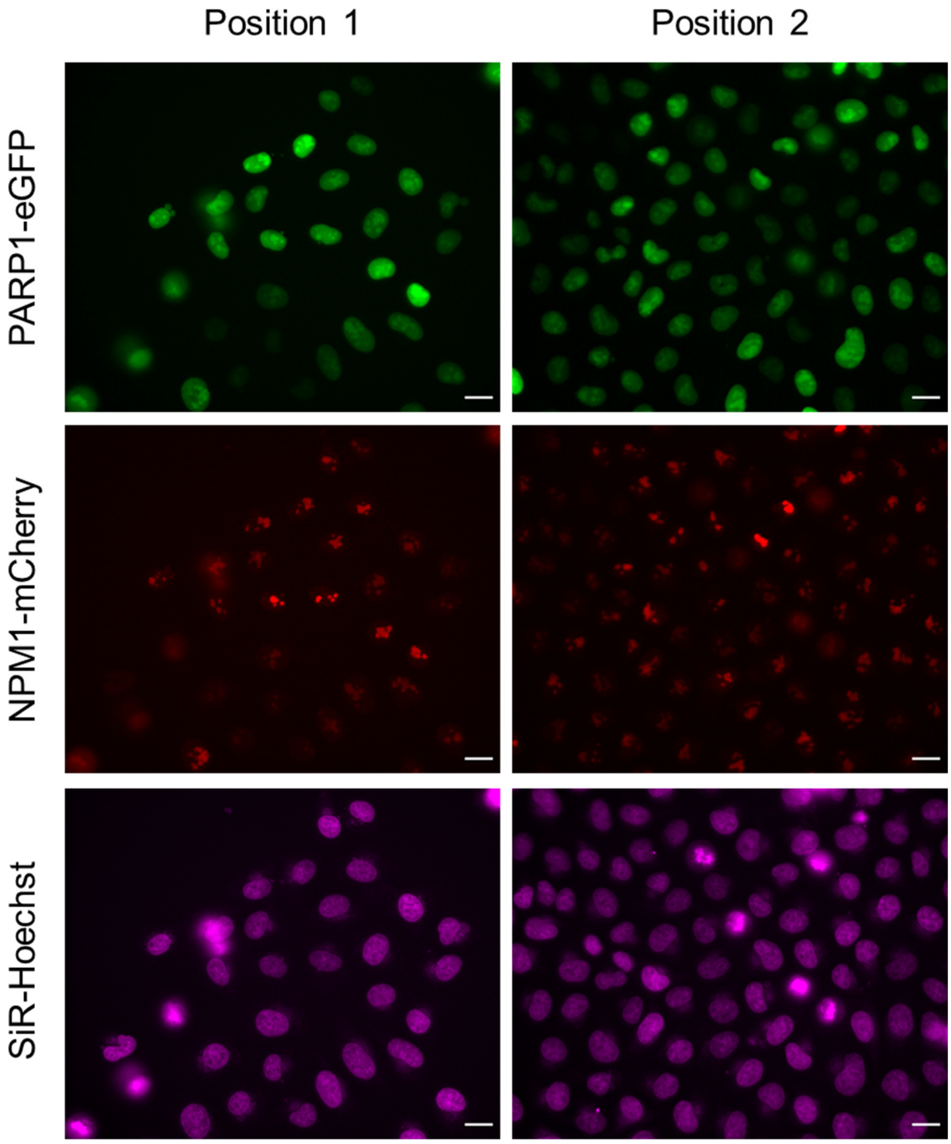

Suppl. Figure 1. Exemplary imaging data utilized for KNIME image analysis. The microscopic images were captured as described in the Materials and Methods section, employing the HeLa 'PARP1-eGFP+NPM1-mCherry' reporter cell line without H_2O_2 treatment. Presented are exemplary images from two distinct microscopic positions. Usually, under each experimental condition, images from five different microscopic positions were acquired, each containing 20-50 cells, leading to the analysis of mostly more than 100 cells per condition. Scale bars represent 20 µm.

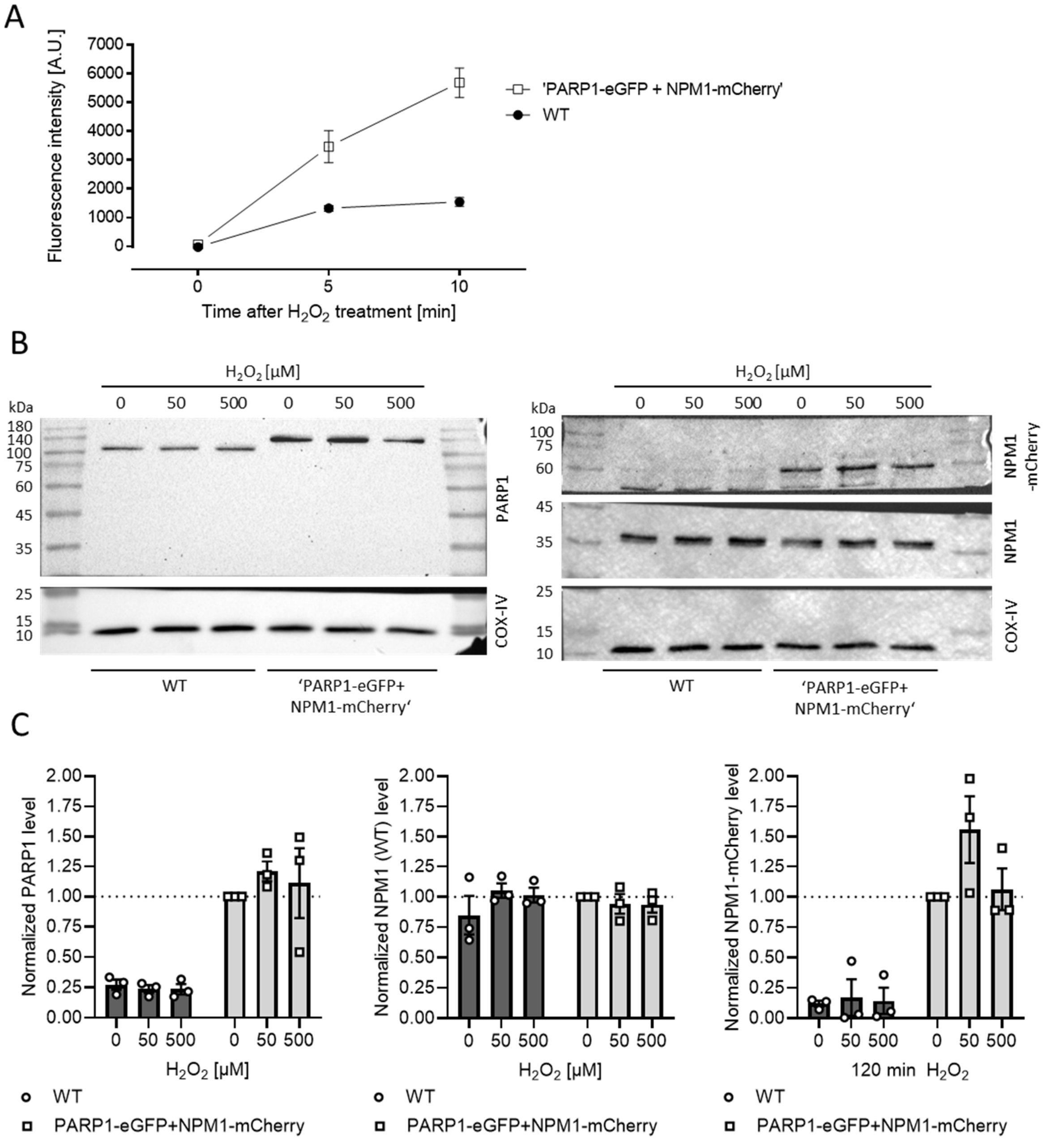

Suppl. Figure 2. Characterization of the HeLa 'PARP1-eGFP+NPM1-mCherry' reporter cell line. (**A**) Analysis of PAR signal intensities of data as shown in **Figure 5.** Signals from at least 40 cells from five microscopic images were evaluated. (**B**) Western blot analyses for PARP1 and NPM1 in HeLa WT and 'PARP1-eGFP+NPM1-mCherry' reporter cells 120 min after treatment with H_2O_2 at concentrations as indicated. Shown are representative blots of three independent experiments. (**C**) Densitometric analyses of signal intensities normalized to the signals of the housekeeper COX-IV and non-treated HeLa 'PARP1-eGFP+NPM1-mCherry' cells. Data represent means ± SEM from three independent biological experiments.

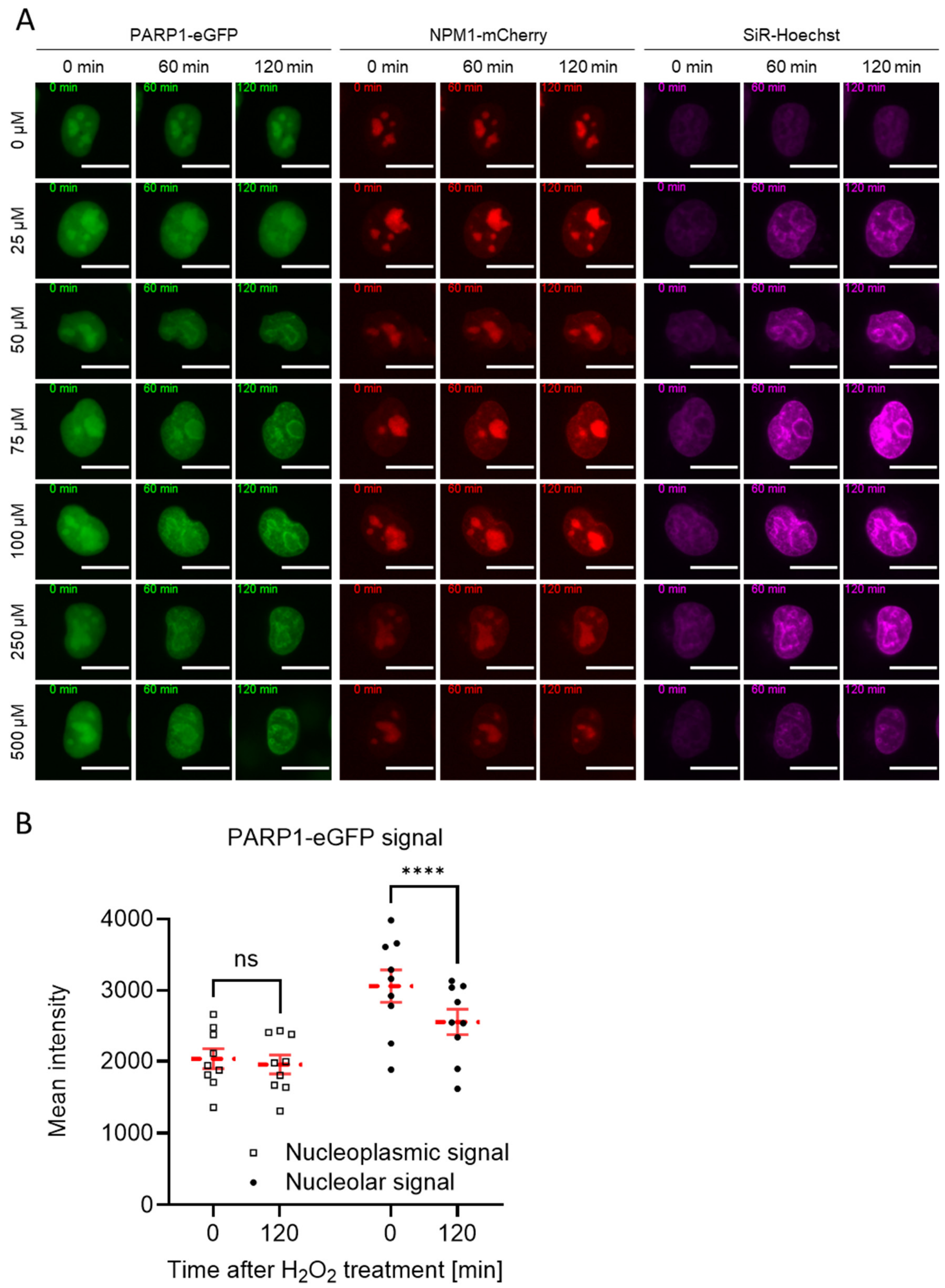

Suppl. Figure 3. PARP1 localization in HeLa 'PARP1-eGFP+NPM1-mCherry' reporter cells upon treatment with H_2O_2. (**A**) Representative magnified images of data shown in **Figure 3** at the time points 0, 60 and 120 min are displayed. For better visibility linear adjustments of brightness and contrast were performed using Fiji. Scale bars represent 10 µm. (**B**) Analysis of nucleoplasmic and nucleolar PARP1-eGFP signal intensities via KNIME before and 120 min after treatment with 50 µM H_2O_2 of data as shown in A.

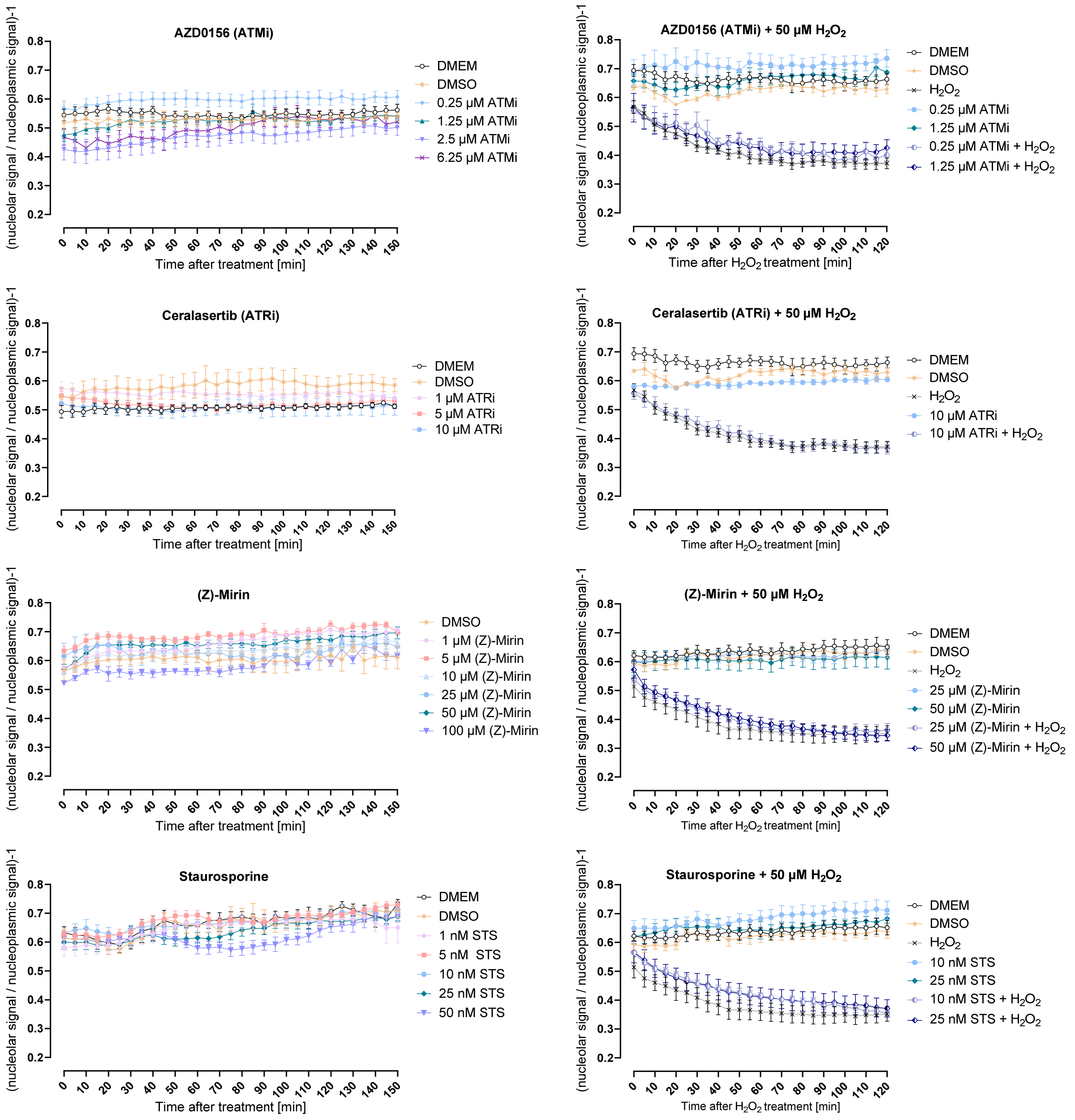

Suppl. Figure 4. Pharmacological screen for potential modulators of H_2O_2-induced nucleolar-nucleoplasmic PARP1 shuttling. HeLa 'PARP1-eGFP + NPM1-mCherry' cells were treated with different pharmacological inhibitors alone (left) or were pre-incubated for 30 min with the respective inhibitor in concentrations as indicated and subsequently treated with 50 µM H_2O_2 (right). Live-cell imaging was started immediately after addition of the inhibitor (left) or immediately after H_2O_2 treatment (right). Image acquisition was performed every 5 min for time periods as indicated. The results of the quantitative image analysis via KNIME are shown. Data represent means ± SEM of five microscopic images typically containing >20 cells per image of one screening experiment. AZD0156 – ATM inhibitor; ceralasertib – ATR inhibitor; (Z)-Mirin – MRN-ATM pathway inhibitor; staurosporine (STS) – broad spectrum kinase inhibitor.

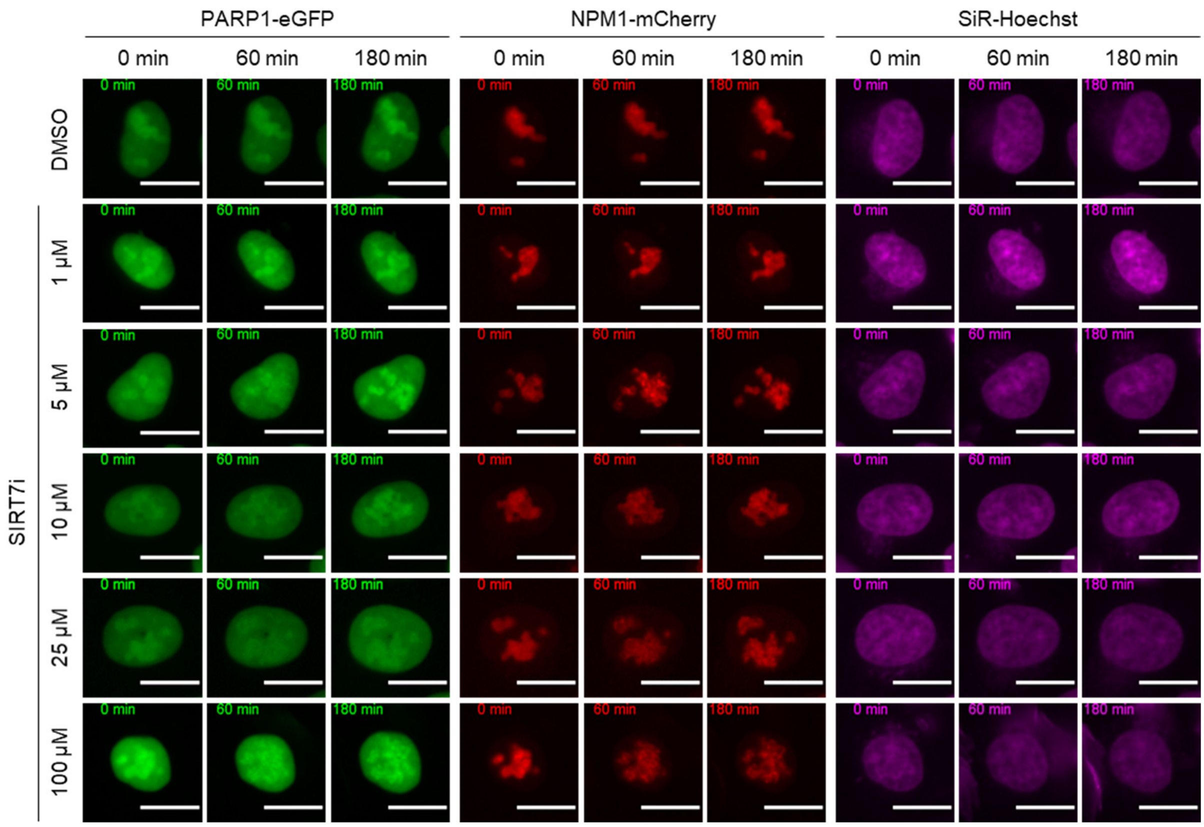

Suppl. Figure 5. PARP1 localization upon SIRT7 inhibition. Representative magnified images of data depicted in **Figure 6** at the time points 0, 60 and 120 min are shown. For better visibility linear adjustments of brightness and contrast were performed using Fiji. Scale bars represent 10 µm.

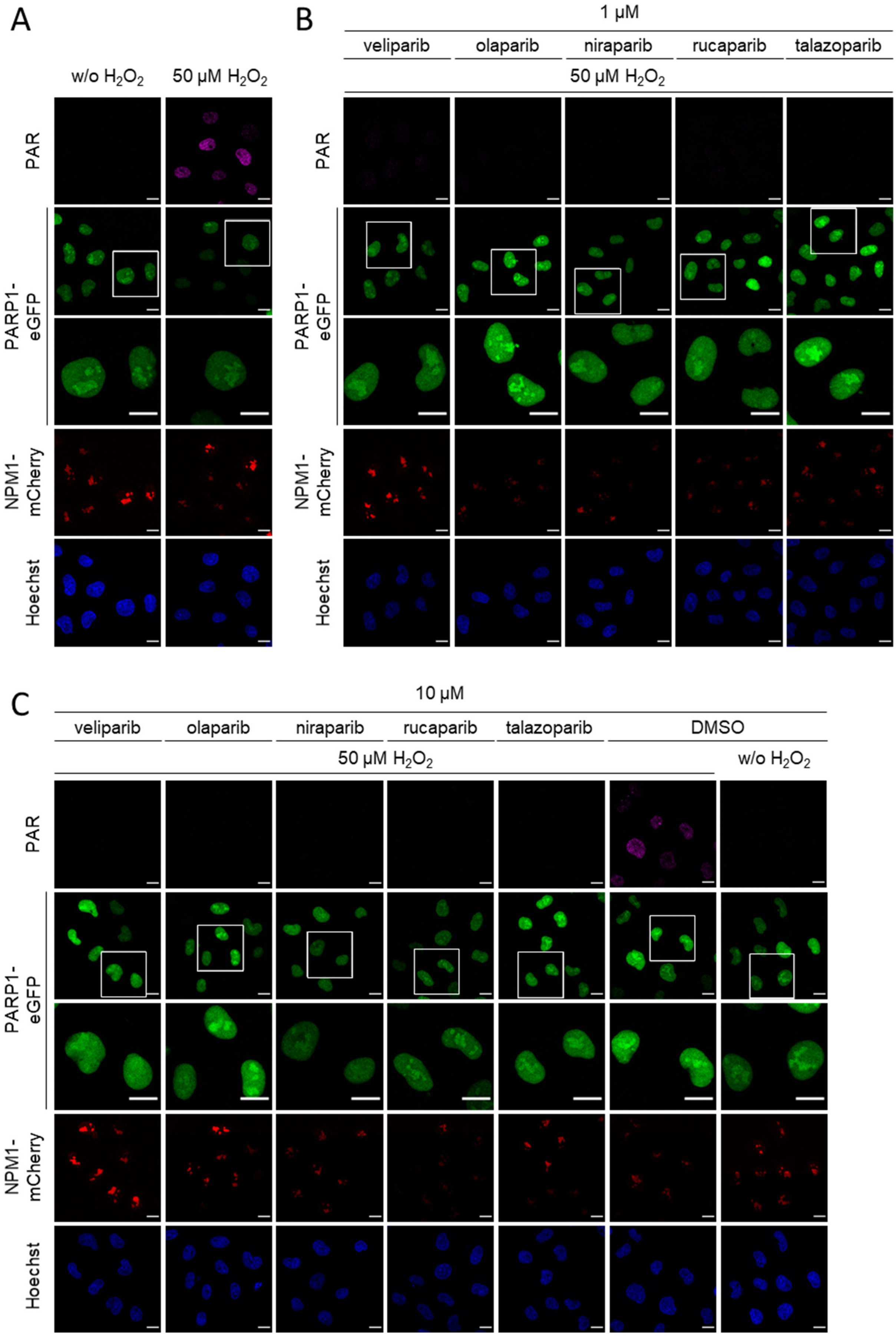
A
w/o H_2O_2
50 µM H_2O_2
PAR
PARP1-eGFP
NPM1-mCherry
Hoechst
B
1 µM
veliparib
olaparib
niraparib
rucaparib
talazoparib
50 µM H_2O_2
PAR
PARP1-eGFP
NPM1-mCherry
Hoechst
C
10 µM
veliparib
olaparib
niraparib
rucaparib
talazoparib
DMSO
50 µM H_2O_2
w/o H_2O_2
PAR
PARP1-eGFP
NPM1-mCherry
Hoechst

Suppl. Figure 6. PARP inhibitor treatments inhibit H_2O_2-induced PARylation in 'PARP1-eGFP + NPM1-mCherry' cells. (**A**) Cells were pre-incubated with (**B**) 1 µM or (**C**) 10 µM of veliparib, rucaparib, olaparib, niraparib or talazoparib, and treated with 50 µM H_2O_2 to induce a PARylation response. As a negative control, cells were mock-treated with DMEM. As a solvent control, cells were pre-incubated with 0.1% (v/v) DMSO. After 5 min of H_2O_2 treatment, cells were fixated, and subjected to immunofluorescence staining for PAR. The Hoechst stain was used to visualize nuclei. Samples were analyzed by confocal microscopy as described in the Material and Methods section. For better visibility linear adjustments of brightness and contrast were performed using Fiji. White frames indicate areas of magnification. Scale bars represent 20 µm.

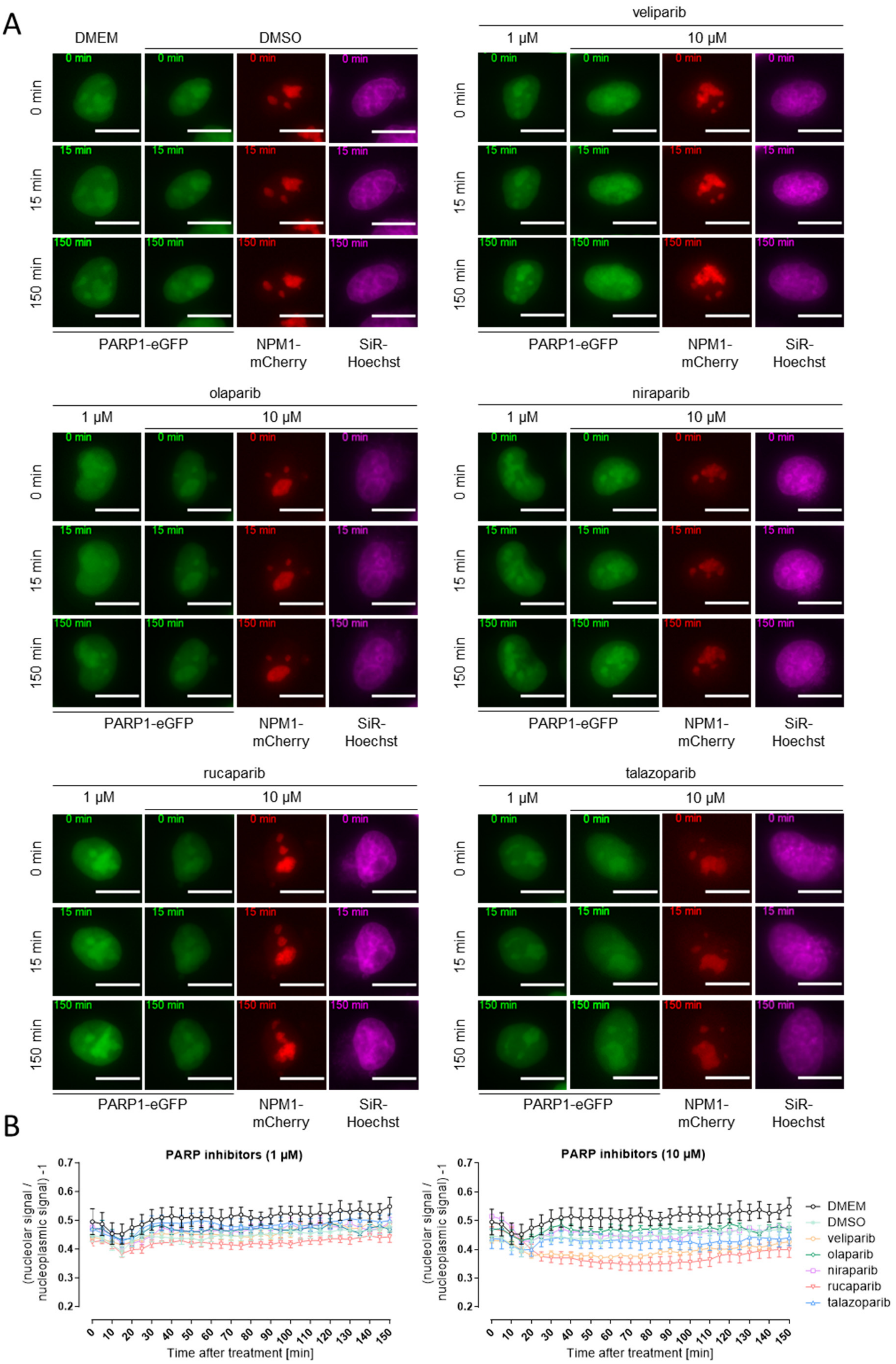
A
DMEM
DMSO
veliparib
olaparib
niraparib
rucaparib
talazoparib
1 μM
10 μM
0 min
15 min
150 min
PARP1-eGFP
NPM1-mCherry
SiR-Hoechst
B
PARP inhibitors (1 μM)
PARP inhibitors (10 μM)
(nucleolar signal / nucleoplasmic signal) -1
Time after treatment [min]
DMEM
DMSO
veliparib
olaparib
niraparib
rucaparib
talazoparib

Suppl. Figure 7. PARP1 localization upon treatment with pharmacological PARP inhibitors. (**A**) Representative magnified images at the time points 0, 15 and 150 min are displayed. For better visibility linear adjustments of brightness and contrast were performed using Fiji. Scale bars represent 10 µm. (**B**) Quantitative automated image analysis via KNIME of data shown in A. Data represent means ± SEM of five microscopic images typically containing >20 cells per image of one experiment.

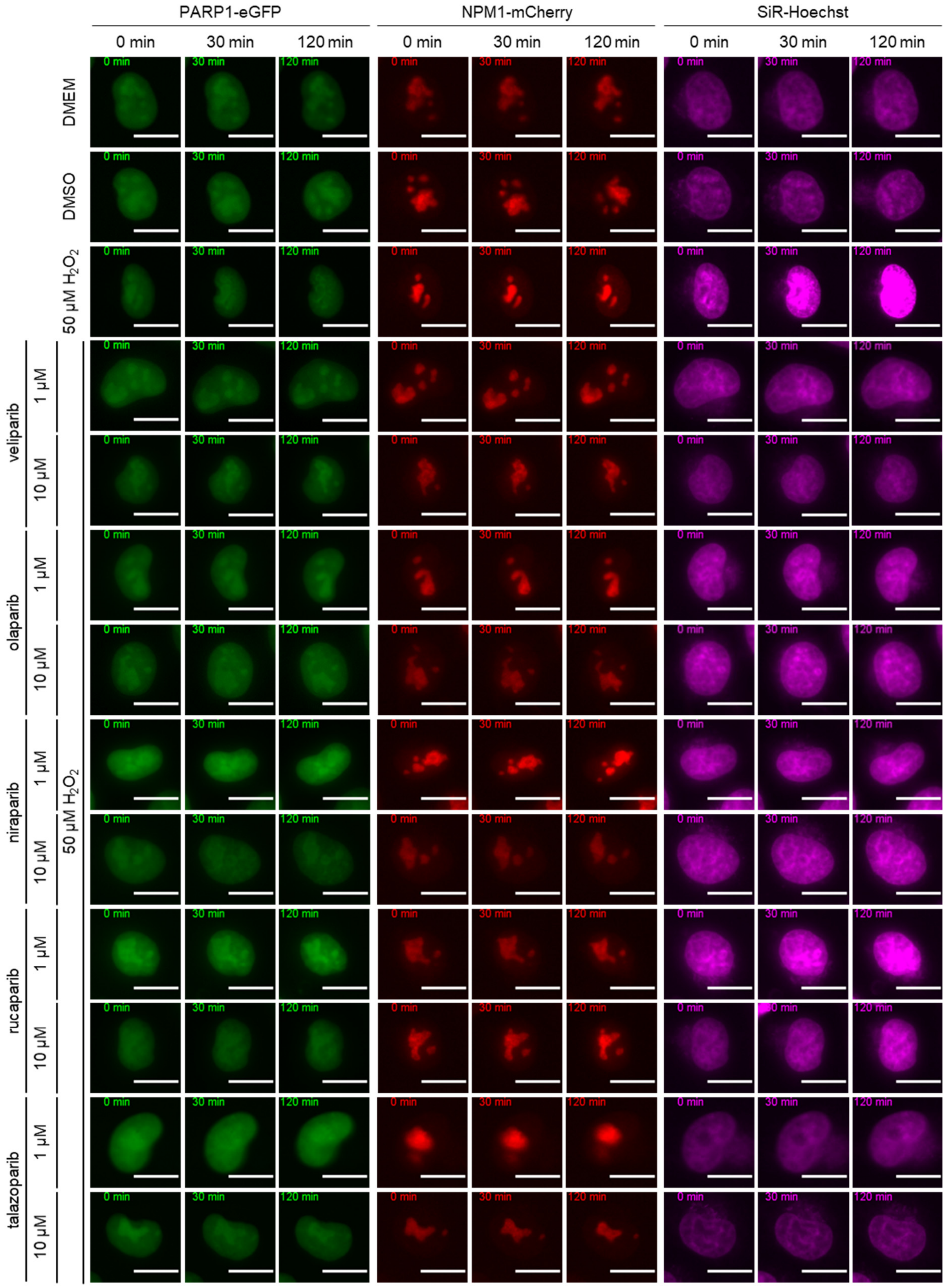

Suppl. Figure 8. PARP1 localization upon co-treatment with pharmacological PARP1 inhibitors and H_2O_2. Representative magnified images of data shown in **Figure 7** and **Suppl. Figure 9** are displayed. For better visibility linear adjustments of brightness and contrast were performed using Fiji. Scale bars represent 10 µm.

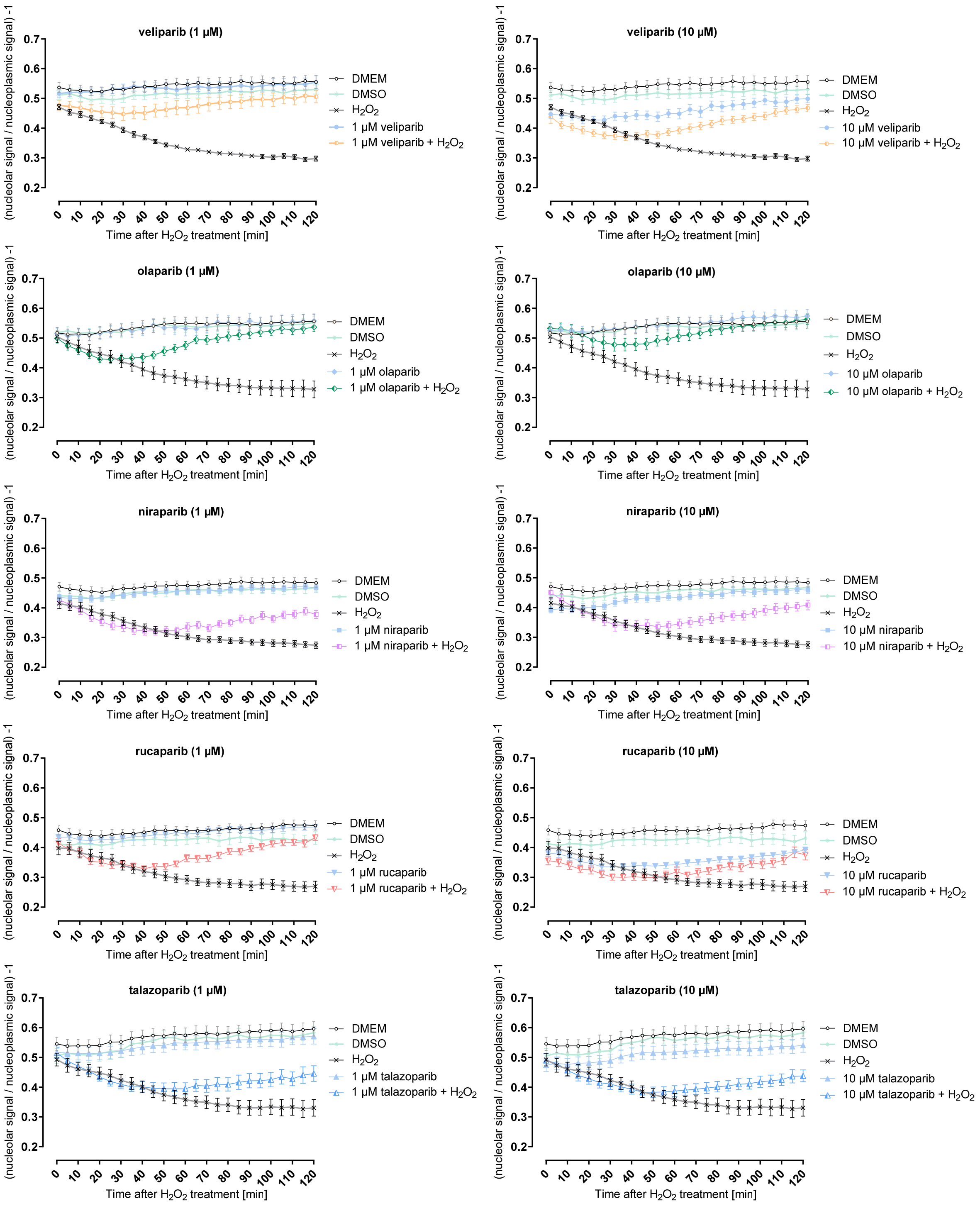

Suppl. Figure 9. PARP1 localization upon co-treatment with pharmacological PARP1 inhibitors and H_2O_2. Alternative presentation of data shown in **Figure 7**. Data represent means ± SEM of five microscopic images per experiment typically containing >20 cells per image of three independent experiment.

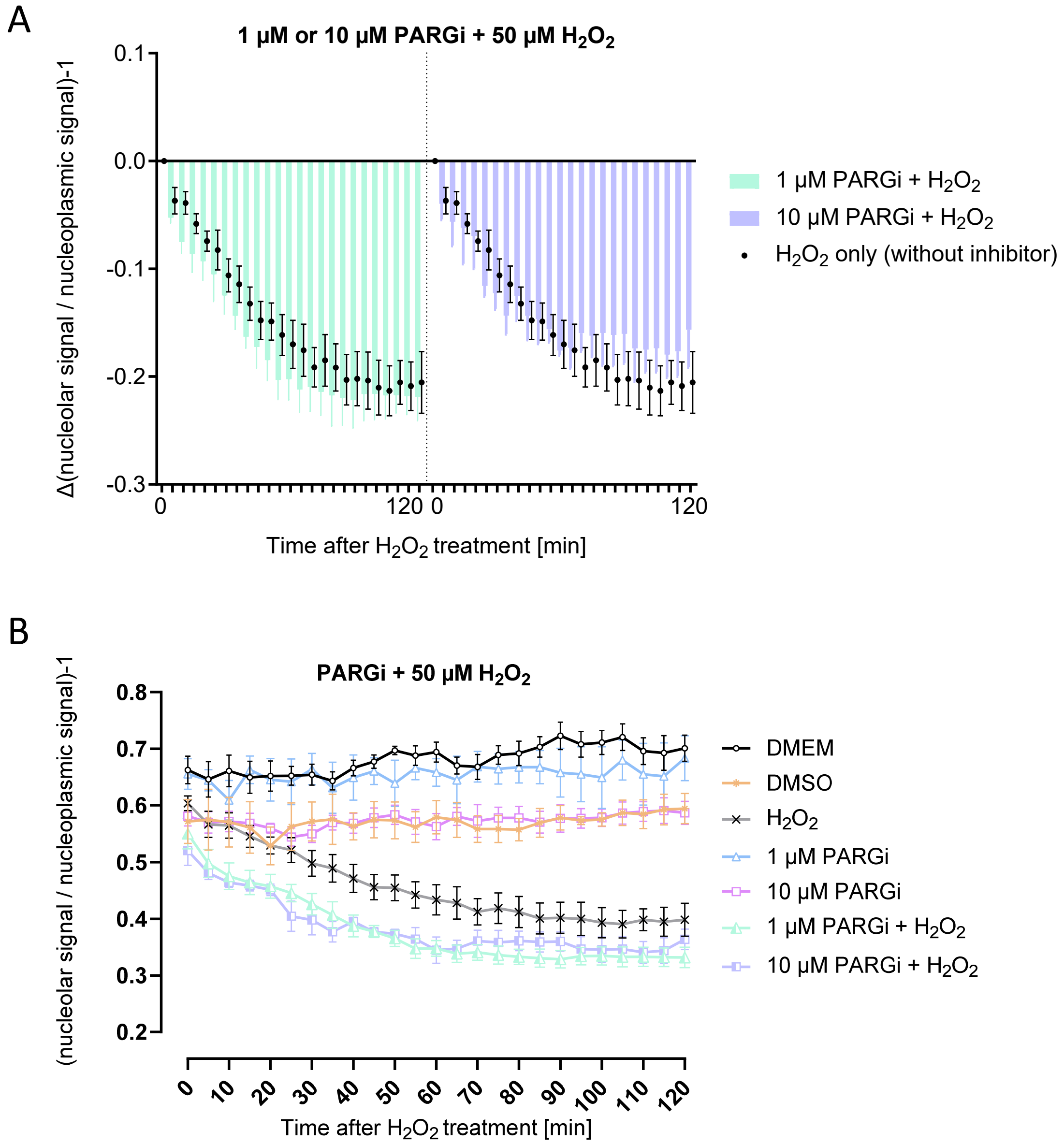

Suppl. Figure 10. PARP1 localization upon PARG inhibition and H_2O_2 treatment. HeLa 'PARP1-eGFP + NPM1-mCherry' cells were pre-incubated for 30 min with concentrations of the PARG inhibitor PDD00017273 as indicated. Afterwards, cells were either mock-treated with DMEM or were treated with 50 µM H_2O_2. Live-cell imaging was started immediately after H_2O_2 treatment and was performed for 120 min in 5 min intervals. (**A**) Depicted are changes in the ratios of PARP1 nucleolar to nucleoplasmic signals as evaluated via KNIME. (**B**) Alternative presentation of data shown in A including controls. Data represent means ± SEM of five microscopic images typically containing >20 cells per image of one experiment.

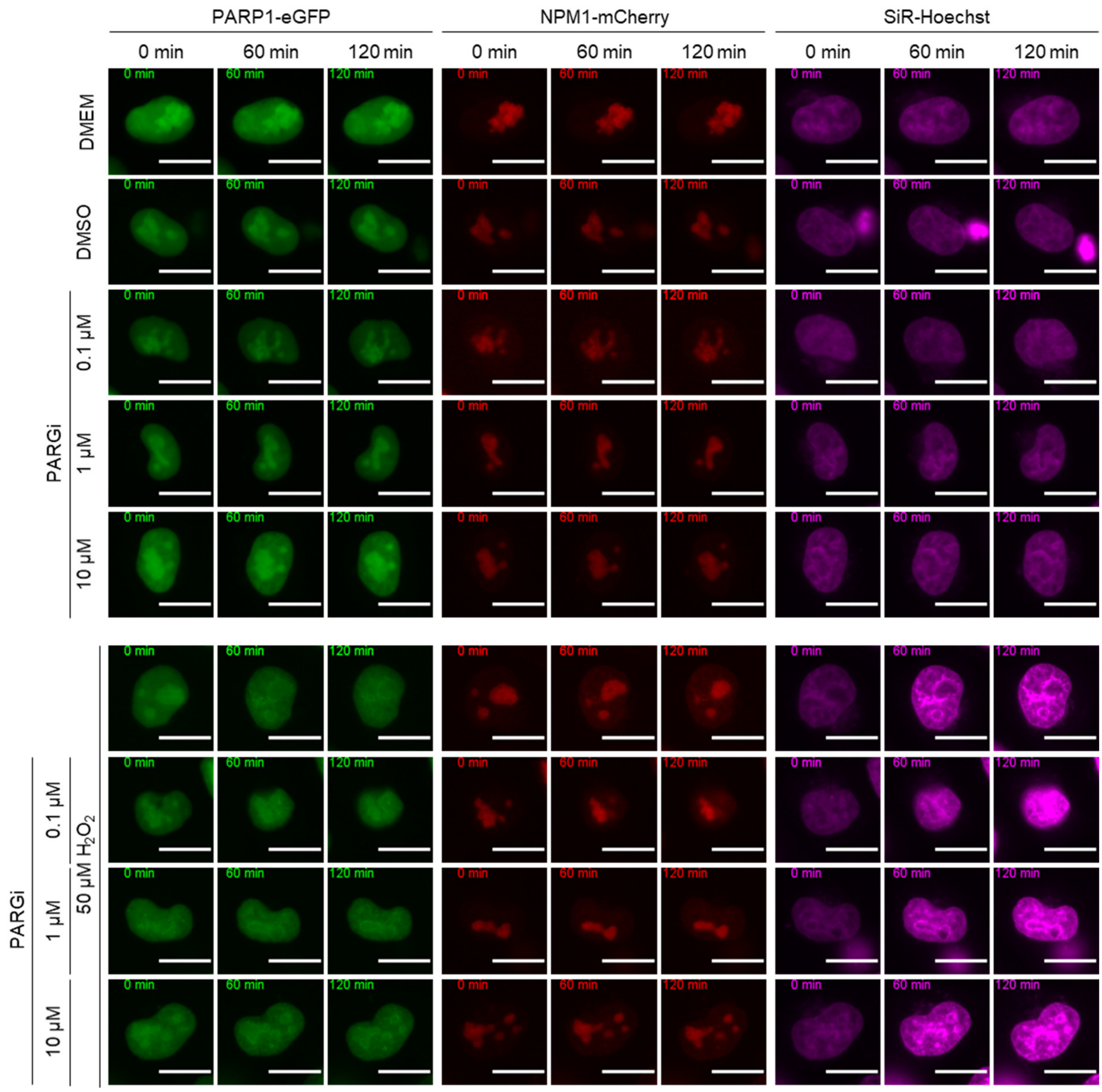

Suppl. Figure 11. PARP1 localization upon co-treatment with the pharmacological PARG inhibitor PDD00017273 and H_2O_2. Representative magnified images of data shown in **Suppl. Figure 10** are displayed. For better visibility linear adjustments of brightness and contrast were performed using Fiji. Scale bars represent 10 µm.

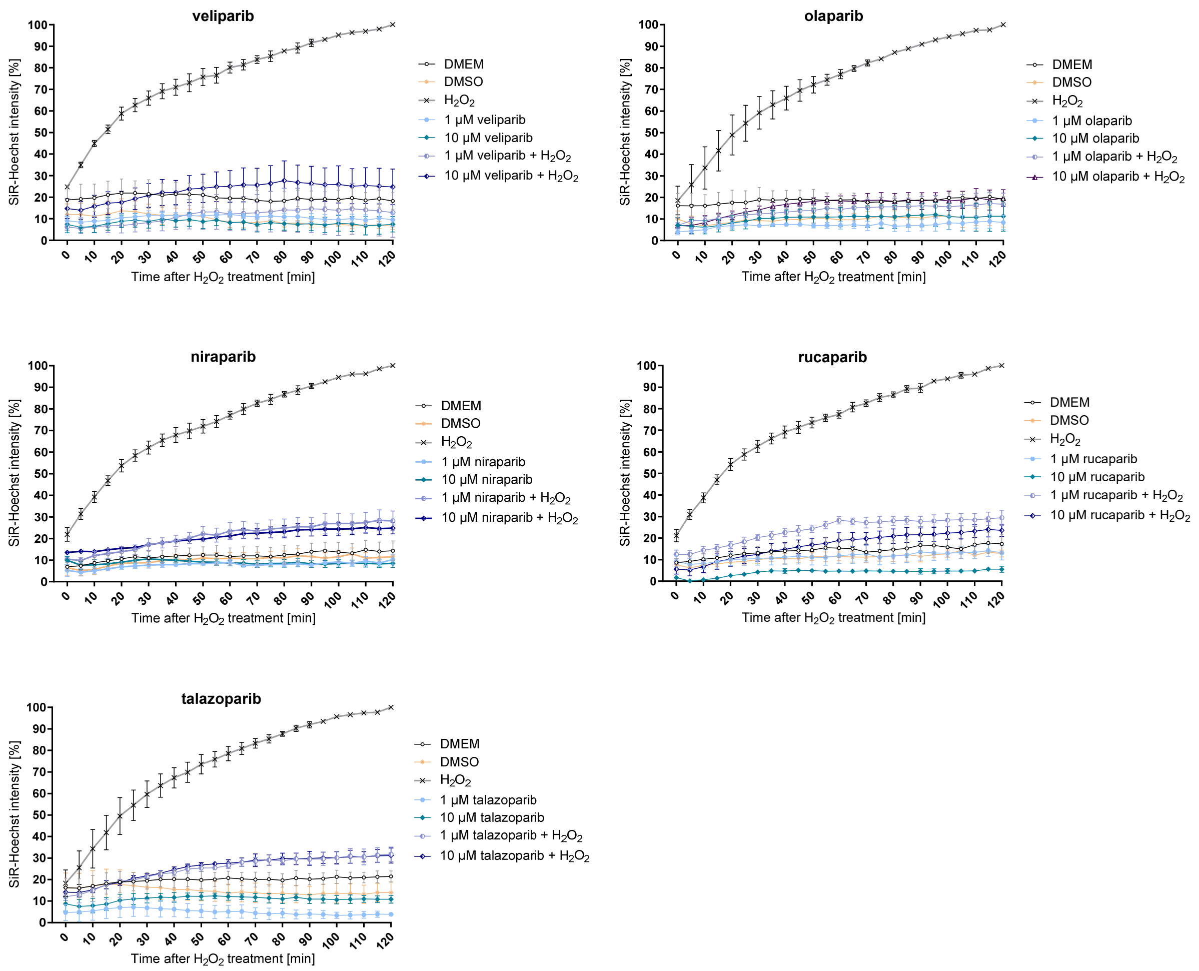

Suppl. Figure 12. H_2O_2-induced increase in SiR-Hoechst intensity is PAR-dependent. HeLa 'PARP1-eGFP + NPM1-mCherry' cells were pre-incubated with 1 or 10 µM of PARPi (i.e., veliparib, olaparib, rucaparib, niraparib or talazoparib) for 30 min and were subsequently treated with 50 µM H_2O_2. The results of the quantitative image analysis via KNIME with regards to SiR-Hoechst intensity of microscopic data as depicted in **Suppl. Figure 8** are shown. The KNIME analysis was performed as described in the Material and Methods section. Data are means ± SEM of three independent experiments.
